# Association between environmental noise and subjective symptoms related to cardiovascular diseases among elderly individuals in Japan

**DOI:** 10.1371/journal.pone.0188236

**Published:** 2017-11-16

**Authors:** Kenichi Azuma, Iwao Uchiyama

**Affiliations:** 1 Department of Environmental Medicine and Behavioral Science, Kindai University Faculty of Medicine, Osakasayama, Osaka, Japan; 2 Sick-house Medical Science Laboratory, Division of Basic Research, Louis Pasteur Center for Medical Research, Kyoto, Japan; CUNY, UNITED STATES

## Abstract

Noise in housing environments may increase the risk of cardiovascular diseases (CVDs); however, the most significant sources of noise among elderly individuals remain poorly understood. A nationwide cross-sectional study comprised of 6,181 elderly people (age ≥ 65 years) was conducted using a web-based self-reported questionnaire in 2014. Questions pertaining to CVD-related subjective symptoms within the past year addressed symptoms of chest pain, disturbances in pulse, acute impaired tongue movement, limb paralysis, and foot pain or numbness during walking. Questions concerning noise included awakening during the night due to noise, automobile, neighborhood, construction, railway, and aircraft noise. The multivariable analyses revealed that all symptoms were significantly associated with awakening during the night due to noise. Automobile, construction, railway, and aircraft noise were significantly associated with more CVD-related symptoms at nighttime than at daytime. Our results suggest that noise at nighttime is an important risk factor for CVDs. Although several different sources of environmental noise, including automobile, neighborhood, construction, railway, and aircraft noise were found to be significantly associated with CVD-related symptoms, the strongest association was observed for construction noise, followed by neighborhood and automobile noise. The adjusted odds ratios (95% confidence intervals) for construction noise at nighttime were 1.12 (1.06–1.19) with disturbances in pulse, 1.21 (1.08–1.35) in acute impaired tongue movement, 1.25 (1.15–1.36) in limb paralysis, and 1.19 (1.12–1.28) in foot pain or numbness during walking. The associations with railway and aircraft noise were found to be weaker than those with automobile, neighborhood, and construction noise. Our study suggests that CVD-related symptoms may exhibit a greater association with construction, neighborhood, and automobile noise than with railway and aircraft noise.

## Introduction

Cardiovascular diseases (CVDs) continue to be the leading cause of death and a major source of health disparities in both Japan and throughout the world [1–3]. Moreover, there has been a rapid increase in CVD-related mortality among elderly individuals [1–3]. Multiple studies have documented associations between risk of CVD and neighborhood socioeconomic characteristics (e.g., diet, smoking, physical activity, psychosocial aspects, and environmental factors) [4]. In addition, environmental noise levels may be related to risk of CVD through the effects of noise on sleep disturbance and possibly stress [4,5]. Furthermore, while some reports have linked exposure to noise with cardiovascular risk factors (e.g., elevated blood pressure and myocardial infarction), evidence of this relationship is mixed [6].

Epidemiological studies have indicated that environmental noise is an important risk factor for CVDs in housing, and indicated that exposure to noise from various sources (e.g., residential neighborhoods, workplaces, road traffic, railway, and aircraft) is associated with CVDs and CVD risk factors, including obesity and hypertension [7–11]. A recent meta-analysis also suggested that individuals over 65 years of age had a higher risk of developing ischemic heart disease (IHD) compared to those under 65 years following exposure to road and aircraft noise [9].

Noise annoyance and the related health problems among residents might be associated with noise emission during the process of building construction, as well as exposed workers [12]; however, the association between CVDs has not been reported to date. Since previous studies have commonly focused on occupational noise sources and transport-related noise; there is a gap that the present study should attempt to address. Therefore, the purpose of this study was to investigate the most significant noise sources associated with CVDs and the CVD risk factors in housing environment among elderly individuals.

## Methods

### Study design and population

To include individuals exposed to various environmental noise sources, a nationwide population-based cross-sectional design was selected. A web-based survey was conducted targeting elderly Japanese subjects (age ≥ 65 years) randomly selected based on demographic data obtained from a web panel of a leading Internet-based research institute in Japan (Macromill Research Panel). An increasing number of population-based epidemiological surveys using a web panel have been reported during the past 10 years and their reliability has been demonstrated [13–16]. Moreover, this research panel has been used in other Japanese epidemiological studies [17–19]. A total of 1,160 thousand members were registered for the web panel as of May 1^st^, 2014, of whom 28,581 were elderly individuals. This research institute has developed a highly credible web panel via thorough quality control and management and planned recruitment. The research institute has periodically evaluated the demographic characteristics of the web panel and has reported that many factors, such as marital status, family structure, annual income, property, alcohol intake, lifestyle, consumption habit, interests, distress, anxiety, life consciousness, and personality, excluding knowledge or use of a computer, considerably corresponded to the results of population-based surveys conducted using traditional sampling measures [20].

This study was approved by the ethical committee for human research at the Louis Pasteur Center for Medical Research (LPC.14). The study was conducted in accordance with the Declaration of Helsinki.

### Data collection

A total of 12,000 members (5,114 males and 6,886 females) were selected from the web panel by stratified random sampling to ensure that the gender ratio matched the Japanese demographic data obtained from the 2010 Population Census [21].

Members of the web panel received an invitation via e-mail to complete the self-reported questionnaire on a website. To prevent unauthorized access by a third party to the website displaying the survey form, the registered e-mail address and passwords were required when participants accessed the website. To avoid information bias, no information on the topic of the questionnaire was provided in the invitation. Inconsistent or incomplete responses to the questionnaire were controlled by a quality control system provided by the research institute. Members responding to the invitation were provided with a link to the website hosting questionnaire, and were directed to complete and transmit their responses. This web-based survey was conducted from May 9^th^ to May 27^th^, 2014 and two reminders were sent to the selected members during the survey.

### Questionnaires

The questionnaire consisted of items concerning personal characteristics, including gender, age, employment status, and body mass index (BMI); lifestyle, including smoking status, drinking habits, regular exercise as physical activity, sufficiency of sleep duration, and depth of sleep; and housing characteristics, including the duration of residence, housing structure, location of the bedroom, proximity of housing to the main road, visibility of the main road, use of an unvented-heating appliance in winter, and the use of incense in the house. The main road was defined in the question as an important road that leads from one town or city to another, such as an expressway, highway, national route, prefectural roads, or municipal road.

The questions regarding health status complied with the Japan Public Health Center-based Prospective Study on Cancer and Cardiovascular Diseases [22]. Questions concerning health outcomes included CVDs under treatment (including those of the parents), such as stroke (cerebral infarction or intracerebral hemorrhage) and IHDs (angina or myocardial infarction). Questions regarding CVD-related subjective symptoms within the past year addressed chest pain: “Have you experienced abrupt chest pain lasting for more than 10 seconds?,” disturbances in pulse: “Have you experienced disturbances in pulse or arrhythmia?,” acute impaired tongue movement: “Have you experienced sudden feeling of thickness of the tongue?,” limb paralysis: “Have you experienced crippled feelings or numb hands and feet?,” and foot pain or numbness during walking: “Have you experienced intermittent claudication in the legs?.”

To evaluate the overall effects of environmental noise on sleeping, the nuisance-related question on environmental noise included awakening during the night due to noise. The source of the noise is not limited in this question. The questions assessing annoyance in response to environmental noise, “Do you feel annoyed by any noise in the home that you are living in now?” included 10 items, including daytime/nocturnal automobile (road traffic), neighborhood (e.g., sound of music, television, radio, bathing, showering, speaking from nearby housing or facilities), vicinal construction, railways, and aircraft noise. The responses regarding perceived annoyance due to environmental noise were rated on a six-point Likert scale ranging from no noise (1) to highly annoyed (6) with reference to the reported noise annoyance scales [23,24]. We added “no noise” to the reported scales for participant not having exposure to environmental noise.

### Statistical analyses

The internal consistency of the perceived noise annoyance scales was evaluated using Cronbach’s alpha. We examined correlations (Spearman’s test) between the variables on the perceived noise annoyance responses for multicollinearity by creating a correlation matrix and scanning for highly correlated variables [25,26].

We used χ^2^ tests to assess the association of the noise factors with potential confounders. To determine the noise factors (predictor variables) associated with CVDs and the related subjective symptoms (dependent variable), noise nuisance and the selected noise annoyance were tested using multiple logistic regression analyses. Confounders that is common causes of both exposure and outcome [27] were selected by reviewing the literature on the noise factors and CVDs [4–11]. Potential confounders included employment status, proximity of housing to the main road, visibility of the main road, location of the bedroom, duration of residence, housing structure, gender, and age. To be comprehensive, we also adjusted for covariates that might impact CVDs but are unlikely to determine noise exposures, including BMI, medical history in parents related to health outcomes, smoking status, drinking habits, regular exercise, sufficiency of sleep duration, depth of sleep, use of an unvented-heating appliance in winter, and use of incense in the house. We built models in stages to examine the effects of measured confounding. Model 1 adjusted for employment status, proximity of housing to the main road, visibility of the main road, location of the bedroom, duration of residence, housing structure, gender, and age. To control for the association with personal characteristics, based on Model 1 we additionally adjusted for variables of BMI and medical history in parents related to health outcomes (Model 2). Finally, to control for the association with lifestyle (smoking status, drinking habits, regular exercise, sufficiency of sleep duration, depth of sleep, use of an unvented-heating appliance in winter, and use of incense in the house), based on Model 2 we additionally adjusted for these variables in Model 3. We used *p* < 0.05 to indicate statistical significance. Odds ratios (ORs) and 95% confidence intervals (CIs) were determined for the univariate and multivariable associations. All data analyses were performed using IBM SPSS version 23 for Windows (IBM Corp, Armonk, NY, USA).

## Results

### Participants

Of the 12,000 members who received an invitation, 6,181 responses (response rate 59.0%) were obtained from 47 prefectures in Japan. For examining the sample of participants on a nationwide scale, correlation test was performed between the number of participants and the Japanese elderly population (age ≥ 65 years) [21] in 47 prefectures in Japan. The Pearson’s correlation coefficient was 0.96 (*p* < 0.001). In addition, the mean proportion of participants to the Japanese elderly population for each of the 47 prefectures was 0.0211% (standard deviation, 0.0087%). Rates of current smoking for every day and sometimes in this study were 10.5% and 1.0%, respectively, and those in the Comprehensive Survey of Living Conditions 2011, Japan [28] were 10.0% and 1.0%, respectively. Rates of drinking habits for once or more a week and less than once a week or never in this study were 47.9% and 52.0%, respectively, and those in the Comprehensive Survey of Living Conditions 2011, Japan [28] were 30.0% and 68.2%, respectively. The prevalence of stroke and IHDs in this study were 2.1% and 4.5%, respectively, and those in the Comprehensive Survey of Living Conditions 2011, Japan [28] were 3.5% and 5.5%, respectively. Similar tendencies were obtained in comparison with the overall elderly population in Japan.

The characteristics of the participants by noise exposure status are presented in Table 1 and the detailed data are shown in supplemental material as S1 Table. The mean age of the subjects was 69.4 years (range: 65–93 years; 51.2% females and 48.8% males). Regarding environmental noise annoyance, the highest proportion of participants responded with construction noise, followed by aircraft, automobile, and neighborhood noise, whereas railway noise had the lowest proportion of responses. The proportion of noise annoyance were higher at nighttime compared with daytime for automobile, neighborhood, and railway noise; however, the inverse relationship was found for construction and aircraft noise.

**Table 1:** Participant characteristics by noise exposure status (*n* = 6181).

	Awakening during the night due to noise (with)	Noise annoyance ^b^ (Scales 4+5+6)									
		Automobile		Neighborhood		Construction		Railway		Aircraft	
		Daytime	Nighttime	Daytime	Nighttime	Daytime	Nighttime	Daytime	Nighttime	Daytime	Nighttime
No. of participants, *n* (%)	1531 (24.8)	430 (7.0)	650 (10.5)	340 (5.5)	477 (7.7)	1140 (18.4)	954 (15.4)	134 (2.2)	180 (2.9)	631 (10.2)	600 (9.7)
Age (years), mean ± SD	69.0 ± 4.0	69.4 ± 4.1	69.4 ± 4.3	68.7 ± 3.5	68.7 ± 3.7	69.3 ± 4.0	69.4 ± 4.1	68.8 ± 4.1	69.2 ± 4.2	69.6 ± 4.3	69.7 ± 4.3
Male, *n* (%)	651 (42.5)	210 (48.8)	332 (51.1)	160 (47.1)	213 (44.7)	545 (47.8)	473 (49.6)	55 (41.0)	87 (48.3)	338 (53.6)	328 (54.7)
Employment, *n* (%)											
Employed	131 (8.6)	28 (6.5)	54 (8.4)	33 (9.8)	42 (8.8)	86 (7.6)	82 (8.6)	10 (7.3)	14 (7.9)	45 (7.1)	47 (7.8)
Self-employed	125 (8.1)	31 (7.2)	50 (7.7)	28 (8.3)	37 (7.8)	103 (9.0)	91 (9.6)	18 (13.4)	22 (12.3)	48 (7.6)	47 (7.8)
Housewife/househusband	566 (37.0)	140 (32.6)	197 (30.3)	109 (32.1)	160 (33.5)	373 (32.7)	296 (31.0)	47 (35.1)	56 (31.1)	183 (29.0)	173 (28.8)
Part-time employed	136 (8.9)	40 (9.3)	59 (9.1)	36 (10.6)	51 (10.7)	98 (8.6)	94 (9.9)	9 (6.7)	9 (5.0)	60 (9.5)	51 (8.5)
Unemployed	450 (29.4)	146 (34.0)	226 (34.8)	102 (30.0)	149 (31.2)	376 (33.0)	307 (32.2)	39 (29.1)	60 (33.3)	230 (36.5)	223 (37.2)
Others	123 (8.0)	45 (10.5)	64 (9.8)	32 (9.4)	38 (8.0)	104 (9.1)	84 (8.8)	11 (8.2)	19 (10.6)	65 (10.3)	59 (9.8)
Degree of obesity, *n* (%) ^a^											
Underweight	129 (8.4)	31 (7.2)	52 (8.0)	18 (5.3)	35 (7.4)	76 (6.7)	54 (5.7)	8 (6.0)	12 (6.7)	48 (7.6)	41 (6.8)
Normal	1128 (73.7)	334 (77.7)	499 (76.9)	264 (77.6)	371 (77.9)	839 (73.7)	710 (74.5)	97 (72.4)	137 (76.1)	462 (73.2)	440 (73.5)
Obesity (class I)	249 (16.3)	56 (13.0)	84 (12.9)	56 (16.5)	67 (14.1)	199 (17.5)	170 (17.8)	27 (20.1)	29 (16.1)	110 (17.4)	109 (18.2)
Obesity (class II)	20 (1.3)	7 (1.6)	12 (1.8)	2 (0.6)	3 (0.6)	22 (1.9)	17 (1.8)	2 (1.5)	2 (1.1)	10 (1.6)	9 (1.5)
Obesity (class III)	5 (0.3)	2 (0.5)	1 (0.2)	0 (0.0)	0 (0.0)	2 (0.2)	1 (0.1)	0 (0.0)	0 (0.0)	1 (0.2)	0 (0.0)
Obesity (class IV)	0 (0.0)	0 (0.0)	1 (0.2)	0 (0.0)	0 (0.0)	1 (0.1)	1 (0.1)	0 (0.0)	0 (0.0)	0 (0.0)	0 (0.0)
Medical history of parents (with), *n* (%)											
Stroke	326 (21.3)	81 (18.8)	140 (21.5)	72 (21.2)	98 (20.5)	237 (20.8)	206 (21.6)	32 (23.9)	46 (25.6)	139 (22.0)	137 (22.8)
IHDs	202 (13.2)	64 (14.9)	93 (14.3)	51 (15.0)	64 (13.4)	157 (13.8)	121 (12.7)	15 (11.2)	21 (11.7)	74 (11.7)	71 (11.8)
Smoking status, *n* (%)											
Current/every day	139 (9.1)	38 (8.8)	67 (10.3)	33 (9.7)	39 (8.2)	128 (11.2)	105 (11.0)	13 (9.7)	15 (8.3)	58 (9.2)	49 (8.2)
Current/sometimes	17 (1.1)	4 (0.9)	8 (1.2)	3 (0.9)	2 (0.4)	11 (1.0)	11 (1.2)	2 (1.5)	6 (3.3)	7 (1.1)	9 (1.5)
Former	476 (31.1)	142 (33.0)	214 (32.9)	110 (32.4)	146 (30.6)	350 (30.7)	285 (29.9)	39 (29.1)	60 (33.3)	236 (37.4)	223 (37.2)
Never	899 (58.7)	246 (57.2)	361 (55.5)	194 (57.1)	290 (60.8)	651 (57.1)	553 (58.0)	80 (59.7)	99 (55.0)	330 (52.3)	319 (53.2)
Drinking habits, *n* (%)											
Once or more a week	731 (47.8)	228 (53.1)	320 (49.3)	170 (50.0)	224 (47.0)	567 (49.8)	475 (49.7)	72 (53.8)	91 (50.5)	311 (49.3)	296 (49.3)
Less than once a week or never	800 (52.3)	202 (47.0)	330 (50.8)	170 (50.0)	253 (53.0)	573 (50.3)	479 (50.2)	62 (46.3)	89 (49.5)	320 (50.7)	304 (50.7)
Regular exercise, *n* (%)											
Current/always	525 (34.3)	161 (37.4)	229 (35.2)	123 (36.2)	168 (35.2)	404 (35.4)	331 (34.7)	40 (29.9)	55 (30.6)	209 (33.1)	202 (33.7)
Current/sometimes	649 (42.4)	174 (40.5)	263 (40.5)	143 (42.1)	207 (43.4)	497 (43.6)	414 (43.4)	63 (47.0)	86 (47.8)	275 (43.6)	255 (42.5)
Former	221 (14.4)	50 (11.6)	81 (12.5)	34 (10.0)	56 (11.7)	138 (12.1)	120 (12.6)	15 (11.2)	18 (10.0)	72 (11.4)	73 (12.2)
Never	136 (8.9)	45 (10.5)	77 (11.8)	40 (11.8)	46 (9.6)	101 (8.9)	89 (9.3)	16 (11.9)	21 (11.7)	75 (11.9)	70 (11.7)
Sufficiency of sleep duration, *n* (%)											
Short	566 (37.0)	152 (35.3)	234 (36.0)	127 (37.4)	167 (35.0)	375 (32.9)	306 (32.1)	49 (36.6)	60 (33.3)	202 (32.0)	187 (31.2)
Average	160 (10.5)	39 (9.1)	74 (11.4)	32 (9.4)	47 (9.9)	121 (10.6)	108 (11.3)	14 (10.4)	24 (13.3)	62 (9.8)	62 (10.3)
Good	764 (49.9)	222 (51.6)	320 (49.2)	166 (48.8)	245 (51.4)	607 (53.2)	507 (53.1)	65 (48.5)	89 (49.4)	342 (54.2)	326 (54.3)
Long	41 (2.7)	17 (4.0)	22 (3.4)	15 (4.4)	18 (3.8)	37 (3.2)	33 (3.5)	6 (4.5)	7 (3.9)	25 (4.0)	25 (4.2)
Depth of sleep, *n* (%)											
Deep	93 (6.1)	45 (10.5)	66 (10.2)	35 (10.3)	51 (10.7)	152 (13.3)	120 (12.6)	19 (14.2)	22 (12.2)	88 (13.9)	70 (11.7)
Fairly deep	686 (44.8)	210 (48.8)	303 (46.6)	158 (46.5)	213 (44.7)	580 (50.9)	477 (50.0)	65 (48.5)	83 (46.1)	307 (48.7)	294 (49.0)
Average	43 (2.8)	13 (3.0)	24 (3.7)	10 (2.9)	11 (2.3)	28 (2.5)	24 (2.5)	5 (3.7)	8 (4.4)	24 (3.8)	24 (4.0)
Fairly light	557 (36.4)	120 (27.9)	196 (30.2)	99 (29.1)	152 (31.9)	299 (26.2)	261 (27.4)	33 (24.6)	55 (30.6)	169 (26.8)	169 (28.2)
Light	152 (9.9)	42 (9.8)	61 (9.4)	38 (11.2)	50 (10.5)	81 (7.1)	72 (7.5)	12 (9.0)	12 (6.7)	43 (6.8)	43 (7.2)
Proximity of housing to the main road, *n* (%)											
Along with road	198 (12.9)	116 (27.0)	153 (23.5)	43 (12.6)	66 (13.8)	165 (14.5)	161 (16.9)	22 (16.4)	25 (13.9)	56 (8.9)	59 (9.8)
≤50 m	281 (18.4)	57 (13.3)	129 (19.8)	53 (15.6)	89 (18.7)	208 (18.2)	192 (20.1)	14 (10.4)	28 (15.6)	111 (17.6)	97 (16.2)
50 to ≤150 m	294 (19.2)	65 (15.1)	99 (15.2)	62 (18.2)	88 (18.4)	224 (19.6)	198 (20.8)	31 (23.1)	44 (24.4)	118 (18.7)	120 (20.0)
150 to ≤500 m	414 (27.0)	100 (23.3)	149 (22.9)	92 (27.1)	116 (24.3)	294 (25.8)	211 (22.1)	32 (23.9)	45 (25.0)	198 (31.4)	176 (29.3)
>500 m	344 (22.5)	92 (21.4)	120 (18.5)	90 (26.5)	118 (24.7)	249 (21.8)	192 (20.1)	35 (26.1)	38 (21.1)	148 (23.5)	148 (24.7)
Visibility of the main road, *n* (%)											
Good	189 (12.3)	95 (22.1)	136 (20.9)	42 (12.4)	54 (11.3)	148 (13.0)	153 (16.0)	28 (20.9)	40 (22.2)	59 (9.4)	58 (9.7)
Partly	394 (25.7)	121 (28.1)	191 (29.4)	84 (24.7)	144 (30.2)	317 (27.8)	284 (29.8)	35 (26.1)	50 (27.8)	147 (23.3)	137 (22.8)
Poor	948 (61.9)	214 (49.8)	323 (49.7)	214 (62.9)	279 (58.5)	675 (59.2)	517 (54.2)	71 (53.0)	90 (50.0)	425 (67.4)	405 (67.5)
Bedroom is close to main road (with), *n* (%)	207 (13.5)	89 (20.7)	130 (20.0)	44 (12.9)	62 (13.0)	185 (16.2)	167 (17.5)	19 (14.2)	27 (15.0)	93 (14.7)	89 (14.8)
Duration of residence, *n* (%)											
<3 years	86 (5.6)	11 (2.6)	18 (2.8)	12 (3.5)	17 (3.6)	32 (2.8)	27 (2.8)	4 (3.0)	5 (2.8)	20 (3.2)	19 (3.2)
3 to <20 years	503 (32.9)	147 (34.2)	212 (32.6)	118 (34.8)	156 (32.7)	353 (31.0)	293 (30.8)	53 (39.6)	71 (39.5)	185 (29.3)	165 (27.5)
≥ 20 years	942 (61.5)	272 (63.2)	420 (64.6)	210 (61.8)	304 (63.8)	755 (66.2)	634 (66.4)	77 (57.5)	104 (57.7)	426 (67.6)	416 (69.4)
Housing structure, *n* (%)											
Wooden	932 (60.9)	266 (61.9)	406 (62.5)	209 (61.5)	260 (54.5)	658 (57.8)	554 (58.1)	73 (54.4)	103 (57.3)	412 (65.3)	397 (66.2)
Reinforced concrete	512 (33.4)	141 (32.8)	215 (33.1)	118 (34.7)	199 (41.8)	419 (36.7)	355 (37.3)	56 (41.8)	72 (40.0)	179 (28.4)	164 (27.4)
Others	87 (5.7)	23 (5.3)	29 (4.5)	13 (3.8)	18 (3.8)	63 (5.5)	45 (4.7)	5 (3.7)	5 (2.8)	40 (6.3)	39 (6.5)
Use of an unvented-heating appliance in winter (with), *n* (%)	724 (47.3)	195 (45.3)	308 (47.4)	149 (43.8)	203 (42.6)	501 (43.9)	429 (45.0)	52 (38.8)	63 (35.0)	300 (47.5)	281 (46.8)
Use of incense in the house (with), *n* (%)	816 (53.3)	241 (56.0)	367 (56.5)	179 (52.6)	249 (52.2)	641 (56.2)	546 (57.2)	71 (53.0)	98 (54.4)	332 (52.6)	317 (52.8)

^a^ Data were missing for four participants.

^b^ The six levels were (1) no noise, (2) not annoyed, (3) less annoyed, (4) moderately annoyed, (5) annoyed, and (6) highly annoyed.

Abbreviations: IHD, ischemic heart diseases; SD, standard deviation.

### Prevalence of CVDs and related symptoms

Table 2 shows the prevalence of CVDs currently under treatment and the related subjective symptoms within the past year. Regarding the subjective symptoms, pulse disturbances had the highest prevalence, followed by foot pain or numbness during walking, chest pain, and limb paralysis, whereas acute impaired tongue movement exhibited the lowest prevalence among the participants. Significantly more men than women reported all CVDs and symptoms of pulse disturbances, limb paralysis, and foot pain or numbness during walking; however, there was no significant relationship for the symptoms of chest pain and acute impaired tongue movement.

**Table 2:** Prevalence of CVDs under treatment and related subjective symptoms within the past year.

	Total, prevalence (95% CI) (*n* = 6,181)	Females, prevalence (95% CI) (*n* = 3,163)	Males, prevalence (95% CI) (*n* = 3,018)
CVDs			
Stroke ***	2.1 (1.7–2.4)	1.0 (0.7–1.4)	3.2 (2.6–3.8)
IHDs ***	4.5 (4.0–5.0)	1.7 (1.2–2.1)	7.5 (6.5–8.4)
CVD-related symptoms			
Chest pain	6.6 (6.0–7.2)	6.5 (5.7–7.4)	6.6 (5.7–7.5)
Disturbances in pulse ***	15.3 (14.4–16.2)	12.5 (11.3–13.6)	18.2 (16.8–19.6)
Acute impaired tongue movement	3.0 (2.6–3.5)	2.7 (2.1–3.2)	3.4 (2.8–4.1)
Limb paralysis ***	5.5 (4.9–6.1)	3.6 (3.0–4.3)	7.5 (6.5–8.4)
Foot pain or numbness during walking ***	10.0 (9.2–10.7)	8.0 (7.1–8.9)	12.0 (10.9–13.2)

Values are expressed as prevalence (95% CI, assumed as a Poisson distribution). Abbreviations: CVD, cardiovascular disease; IHD, ischemic heart diseases.

*** Significant difference between males and females at *p* < 0.001 by Fisher’s exact test.

### Risk factors associated with CVDs and related symptoms

The Cronbach’s alpha coefficient for the perceived environmental noise annoyance scales was 0.938. The Cronbach’s alpha coefficients excluding each item ranged from 0.933 to 0.938. Moreover, the Cronbach’s alpha coefficients were high (range: 0.85–0.96) for all scales. We examined the correlations among the 10 variables pertaining to sources of environmental noise annoyance (data shown in S2 Table). Correlations between daytime and nighttime noises for all types of the sources of environmental noise annoyance were high (correlation ≥ 0.7).

The univariate associations between the CVD-related symptoms and environmental noise annoyances are listed in Table 3. The results of multiple logistic regression analysis models for the association with CVDs and related symptoms are presented in Table 4. No significant association was observed between sources of environmental noise annoyance and CVDs under treatment. All CVD-related symptoms were significantly associated with awakening during the night due to noise in all models.

**Table 3:** Univariate analysis of the association between CVDs, noise nuisance, and environmental noise annoyances.

	CVDs		CVD-related symptoms within the past year				
	Stroke	IHDs	Chest pain	Disturbances in pulse	Acute impaired tongue movement	Limb paralysis	Foot pain or numbness during walking
Awakening during the night due to noise (with)	1.18 (0.80–1.74)	1.22 (0.93–1.59)	1.68 (1.36–2.08)***	1.43 (1.23–1.67)***	1.81 (1.34–2.45)***	1.44 (1.14–1.82)**	1.57 (1.31–1.88)***
Noise annoyance ^a^							
Automobile (d)	1.01 (0.84–1.22)	1.02 (0.89–1.15)	1.18 (1.07–1.32)**	1.10 (1.03–1.19)**	1.10 (0.94–1.28)	1.10 (0.98–1.24)	1.11 (1.01–1.21)*
Automobile (n)	1.01 (0.85–1.20)	1.07 (0.95–1.20)	1.17 (1.06–1.29)**	1.14 (1.07–1.22)***	1.15 (1.00–1.33)	1.19 (1.07–1.33)**	1.14 (1.05–1.23)**
Neighborhood (d)	1.04 (0.87–1.25)	0.93 (0.82–1.06)	1.15 (1.04–1.27)**	1.06 (0.99–1.14)	1.25 (1.08–1.44)**	1.28 (1.14–1.42)***	1.25 (1.14–1.36)***
Neighborhood (n)	1.04 (0.88–1.23)	0.93 (0.82–1.05)	1.11 (1.01–1.22)*	1.05 (0.98–1.13)	1.16 (1.01–1.33)*	1.25 (1.13–1.38)***	1.22 (1.13–1.32)***
Construction (d)	0.92 (0.79–1.07)	0.99 (0.89–1.09)	1.15 (1.06–1.25)***	1.13 (1.06–1.19)***	1.16 (1.03–1.30)*	1.26 (1.15–1.38)***	1.22 (1.14–1.31)***
Construction (n)	0.94 (0.81–1.08)	0.97 (0.88–1.07)	1.09 (1.01–1.18)*	1.13 (1.07–1.20)***	1.25 (1.12–1.40)***	1.27 (1.17–1.38)***	1.21 (1.14–1.29)***
Railway (d)	0.90 (0.73–1.11)	1.02 (0.89–1.17)	1.15 (1.03–1.28)*	0.99 (0.92–1.07)	1.16 (1.00–1.36)	1.12 (0.99–1.26)	1.04 (0.95–1.14)
Railway (n)	0.98 (0.80–1.19)	1.04 (0.91–1.18)	1.16 (1.04–1.28)**	1.01 (0.94–1.09)	1.17 (1.00–1.35)*	1.14 (1.02–1.28)*	1.04 (0.95–1.14)
Aircraft (d)	0.97 (0.83–1.14)	1.05 (0.95–1.16)	1.12 (1.03–1.22)*	1.09 (1.02–1.15)**	1.07 (0.94–1.21)	1.12 (1.02–1.22)*	1.10 (1.02–1.18)**
Aircraft (n)	1.03 (0.89–1.20)	1.11 (1.00–1.22)	1.12 (1.03–1.22)**	1.10 (1.03–1.16)**	1.08 (0.95–1.22)	1.15 (1.05–1.25)**	1.11 (1.03–1.19)**

Values are expressed as crude odds ratios (95% CI) for 6,181 participants with complete data.

Significant at

* *p* < 0.05.

** *p* < 0.01.

*** *p* < 0.001.

^a^ ORs and 95% CIs for six-level linear variables were calculated using one unit of change. The six levels were (1) no noise, (2) not annoyed, (3) less annoyed, (4) moderately annoyed, (5) annoyed, and (6) highly annoyed.

Abbreviations: CVD, cardiovascular disease; IHD, ischemic heart diseases; d, daytime; n, nighttime.

**Table 4:** Multivariable regression analysis for the association between CVDs, noise nuisance, and environmental noise annoyances.

	CVDs		CVD-related symptoms within the past year				
	Stroke	IHDs	Chest pain	Disturbances in pulse	Acute impaired tongue movement	Limb paralysis	Foot pain or numbness during walking
Awakening during the night due to noise (with)							
Model 1	1.33 (0.90–1.97)	1.43 (1.09–1.88)*	1.69 (1.37–2.10)***	1.51 (1.30–1.77)***	1.85 (1.36–2.51)***	1.57 (1.23–1.99)***	1.67 (1.40–2.01)***
Model 2	1.32 (0.89–1.96)	1.39 (1.05–1.83)*	1.67 (1.35–2.07)***	1.50 (1.28–1.75)***	1.86 (1.37–2.52)***	1.56 (1.23–1.99)***	1.68 (1.40–2.02)***
Model 3	1.17 (0.78–1.78)	1.33 (0.99–1.78)	1.42 (1.13–1.79)**	1.31 (1.11–1.54)**	1.42 (1.02–1.96)*	1.31 (1.01–1.68)*	1.46 (1.20–1.77)***
Noise annoyance ^a^							
Automobile (d)							
Model 1	1.04 (0.86–1.25)	1.07 (0.94–1.21)	1.22 (1.09–1.35)***	1.14 (1.06–1.23)***	1.11 (0.95–1.29)	1.12 (0.99–1.25)	1.11 (1.02–1.22)*
Model 2	1.05 (0.87–1.27)	1.07 (0.94–1.22)	1.22 (1.09–1.35)***	1.14 (1.06–1.23)***	1.12 (0.96–1.31)	1.12 (1.00–1.26)	1.12 (1.03–1.23)*
Model 3	1.01 (0.83–1.22)	1.06 (0.93–1.21)	1.17 (1.05–1.31)**	1.10 (1.02–1.19)*	1.04 (0.89–1.22)	1.07 (0.95–1.21)	1.08 (0.99–1.19)
Automobile (n)							
Model 1	1.01 (0.85–1.21)	1.10 (0.98–1.24)	1.20 (1.08–1.32)***	1.17 (1.09–1.26)***	1.15 (1.00–1.33)	1.20 (1.08–1.34)**	1.14 (1.04–1.24)**
Model 2	1.02 (0.85–1.21)	1.10 (0.98–1.25)	1.20 (1.08–1.32)***	1.17 (1.09–1.26)***	1.16 (1.00–1.34)*	1.20 (1.08–1.34)**	1.14 (1.05–1.24)**
Model 3	0.98 (0.82–1.17)	1.09 (0.96–1.23)	1.15 (1.04–1.27)**	1.13 (1.05–1.21)***	1.08 (0.93–1.25)	1.15 (1.03–1.28)*	1.10 (1.01–1.20)*
Neighborhood (d)							
Model 1	1.09 (0.91–1.31)	0.99 (0.87–1.13)	1.16 (1.04–1.28)**	1.08 (1.01–1.16)*	1.26 (1.09–1.46)**	1.32 (1.18–1.47)***	1.28 (1.18–1.40)***
Model 2	1.09 (0.91–1.32)	1.00 (0.88–1.14)	1.16 (1.04–1.28)**	1.08 (1.01–1.17)*	1.27 (1.09–1.47)**	1.32 (1.18–1.48)***	1.29 (1.18–1.40)***
Model 3	1.06 (0.88–1.27)	0.99 (0.86–1.13)	1.12 (1.01–1.24)*	1.05 (0.97–1.13)	1.20 (1.03–1.39)*	1.28 (1.14–1.43)***	1.25 (1.15–1.37)***
Neighborhood (n)							
Model 1	1.09 (0.92–1.29)	0.99 (0.88–1.12)	1.12 (1.02–1.24)*	1.08 (1.01–1.15)*	1.17 (1.02–1.35)*	1.28 (1.16–1.42)***	1.26 (1.16–1.36)***
Model 2	1.10 (0.92–1.30)	1.00 (0.88–1.13)	1.12 (1.01–1.23)*	1.08 (1.00–1.15)*	1.18 (1.03–1.35)*	1.29 (1.16–1.43)***	1.27 (1.17–1.37)***
Model 3	1.06 (0.89–1.26)	0.98 (0.87–1.12)	1.08 (0.98–1.20)	1.04 (0.97–1.12)	1.11 (0.97–1.28)	1.25 (1.12–1.38)***	1.23 (1.14–1.34)***
Construction (d)							
Model 1	0.94 (0.81–1.09)	1.02 (0.92–1.13)	1.17 (1.07–1.27)***	1.15 (1.08–1.21)***	1.17 (1.03–1.31)*	1.28 (1.17–1.40)***	1.24 (1.16–1.33)***
Model 2	0.94 (0.81–1.09)	1.02 (0.92–1.13)	1.16 (1.07–1.26)***	1.14 (1.08–1.21)***	1.17 (1.03–1.32)*	1.28 (1.17–1.40)***	1.24 (1.16–1.33)***
Model 3	0.92 (0.79–1.07)	1.02 (0.92–1.13)	1.14 (1.05–1.24)**	1.12 (1.06–1.19)***	1.12 (0.99–1.26)	1.25 (1.14–1.37)***	1.22 (1.13–1.31)***
Construction (n)							
Model 1	0.94 (0.81–1.09)	0.98 (0.89–1.09)	1.12 (1.02–1.20)*	1.15 (1.09–1.21)***	1.25 (1.12–1.40)***	1.28 (1.17–1.39)***	1.22 (1.14–1.30)***
Model 2	0.95 (0.82–1.10)	0.98 (0.88–1.09)	1.10 (1.02–1.20)*	1.15 (1.09–1.21)***	1.26 (1.13–1.41)***	1.28 (1.18–1.39)***	1.22 (1.14–1.30)***
Model 3	0.92 (0.80–1.07)	0.98 (0.88–1.08)	1.08 (1.00–1.17)	1.12 (1.06–1.19)***	1.21 (1.08–1.35)**	1.25 (1.15–1.36)***	1.19 (1.12–1.28)***
Railway (d)							
Model 1	0.92 (0.74–1.13)	1.05 (0.91–1.21)	1.15 (1.03–1.28)*	0.99 (0.92–1.08)	1.17 (1.00–1.37)*	1.12 (0.99–1.26)	1.04 (0.95–1.15)
Model 2	0.93 (0.75–1.15)	1.06 (0.92–1.22)	1.15 (1.03–1.29)*	1.00 (0.92–1.08)	1.18 (1.01–1.38)*	1.12 (1.00–1.27)	1.05 (0.95–1.15)
Model 3	0.91 (0.73–1.13)	1.05 (0.91–1.22)	1.14 (1.02–1.27)*	0.99 (0.91–1.07)	1.15 (0.98–1.34)	1.11 (0.98–1.25)	1.03 (0.94–1.13)
Railway (n)							
Model 1	0.99 (0.81–1.21)	1.06 (0.92–1.21)	1.15 (1.04–1.28)**	1.01 (0.94–1.09)	1.17 (1.01–1.36)*	1.14 (1.02–1.28)*	1.04 (0.95–1.14)
Model 2	1.00 (0.82–1.22)	1.07 (0.94–1.23)	1.16 (1.04–1.29)**	1.01 (0.94–1.10)	1.18 (1.02–1.37)*	1.14 (1.02–1.28)*	1.05 (0.96–1.15)
Model 3	0.98 (0.80–1.20)	1.07 (0.93–1.22)	1.15 (1.03–1.27)*	1.00 (0.93–1.09)	1.15 (0.99–1.34)	1.13 (1.01–1.27)*	1.03 (0.94–1.13)
Aircraft (d)							
Model 1	0.94 (0.80–1.10)	1.00 (0.89–1.11)	1.12 (1.03–1.22)*	1.07 (1.00–1.13)*	1.06 (0.93–1.20)	1.10 (1.00–1.20)	1.09 (1.01–1.17)*
Model 2	0.94 (0.80–1.10)	1.00 (0.89–1.11)	1.12 (1.03–1.22)*	1.07 (1.01–1.14)*	1.06 (0.93–1.20)	1.09 (1.00–1.20)	1.09 (1.01–1.17)*
Model 3	0.92 (0.79–1.09)	0.99 (0.89–1.11)	1.10 (1.01–1.20)*	1.05 (0.99–1.12)	1.03 (0.91–1.16)	1.07 (0.97–1.18)	1.07 (0.99–1.15)
Aircraft (n)							
Model 1	0.99 (0.85–1.15)	1.05 (0.95–1.17)	1.12 (1.03–1.22)**	1.08 (1.02–1.14)*	1.06 (0.94–1.20)	1.12 (1.03–1.23)*	1.09 (1.02–1.18)*
Model 2	0.99 (0.85–1.16)	1.05 (0.95–1.17)	1.12 (1.03–1.22)**	1.08 (1.02–1.15)*	1.06 (0.94–1.20)	1.12 (1.03–1.23)*	1.10 (1.02–1.18)*
Model 3	0.98 (0.84–1.14)	1.05 (0.94–1.17)	1.10 (1.01–1.20)*	1.06 (1.00–1.13)	1.03 (0.91–1.17)	1.10 (1.00–1.21)*	1.08 (1.00–1.16)*

Values are expressed as adjusted odds ratios (95% CI) for 6,181 participants with complete data in Model 1 and 6,177 participants with complete data in Model 2 and Model 3 because of four missing data in BMI.

Significant at

* *p* < 0.05.

** *p* < 0.01.

*** *p* < 0.001.

^a^ ORs and 95% CIs for six-level linear variables were calculated using one unit of change. The six levels were (1) no noise, (2) not annoyed, (3) less annoyed, (4) moderately annoyed, (5) annoyed, and (6) highly annoyed.

Abbreviations: CVD, cardiovascular disease; IHD, ischemic heart diseases; d, daytime; n, nighttime. Model 1: single predictor variable of noise nuisance or environmental noise annoyances, adjusted for employment status, proximity of housing to the main road, visibility of the main road, location of the bedroom, duration of residence, housing structure, gender, and age; Model 2: Model 1 + BMI and medical history in parents related to health outcomes; Model 3: Model 2 + smoking status, drinking habits, regular exercise, sufficiency of sleep duration, depth of sleep, use of an unvented-heating appliance in winter, and use of incense in the house.

In the associations between noise sources and CVD-related symptoms in the final model, automobile noise was significantly associated with chest pain and disturbances in pulse at both daytime and nighttime. Automobile noise was significantly associated with limb paralysis and foot pain or numbness during walking at nighttime, whereas the associations were not significant at daytime. Neighborhood noise was significantly associated with limb paralysis and foot pain or numbness during walking at both daytime and nighttime. Neighborhood noise was significantly associated with chest pain and acute impaired tongue movement at daytime, whereas the associations were not significant at the nighttime. Construction noise was significantly associated with disturbances in pulse, limb paralysis, and foot pain or numbness during walking at both daytime and nighttime. Besides, the association between construction noise and chest pain at daytime was significant. The association between construction noise and acute impaired tongue movement was also significant at nighttime. Railway and aircraft noise were significantly associated with chest pain at both daytime and nighttime. Railway and aircraft noise were significantly associated with limb paralysis at nighttime. In addition, aircraft noise was significantly associated with foot pain or numbness during walking at nighttime. However, other symptoms were not significantly associated with railway and aircraft noise.

## Discussion

This study was based on a large nationally sample with extensive individual information among elderly individuals, which allowed for better control of known cardiovascular risk and/or modifying factors. Our findings suggested that relationships between sources of environmental noise annoyance and CVDs under treatment were not significant. This may be due to the time difference between the current exposure to noise and the onset of CVDs.

On the other hand, our findings also suggested relationships between sources of environmental noise annoyance and CVD-related symptoms within the past year. Although a significant association was observed for several different sources of environmental noise, including automobile, neighborhood, construction, railway, and aircraft noise, the strongest association was found for construction noise at both daytime and nighttime, followed by neighborhood noise at daytime and automobile noise at nighttime. The associations with railway and aircraft noise were weaker than those with construction, neighborhood, and automobile noise. Automobile, construction, railway, and aircraft noise were significantly associated with more CVD-related symptoms at nighttime than at daytime. All CVD-related symptoms were significantly associated with awakening during the night due to noise. Our results suggest that noise at nighttime is a significant risk factor for CVDs, as suggested by previous reports [5,11].

Multiple previous studies have reported the association with transportation noise [9,11,29,30]. Nevertheless, our findings suggest that vicinal construction noise was the most significant noise among elderly individuals in our study. In China, 42.1% of environmental complaints have been reported to be associated with noise, 64.4%, 25.0%, 7.4%, and 2.2% of which were attributed to neighborhood noise, construction noise, industrial noise, and traffic noise, respectively; thus, noise is becoming a more serious problem due to rapid urbanization throughout China [31]. In our study, regarding environmental noise annoyance, the highest proportion of participants responded with construction noise, followed by aircraft, automobile, and neighborhood noise. Construction activities or projects (e.g., redevelopment or renewal) at a local site in the city are progressing on a daily basis, and are not limited to large-scale urbanization or city development. Therefore, construction noise is a common environmental threat for public health in many countries or local regions.

The majority of local residents who had nearby construction activities in the Western Iran reported high levels of noise annoyance [12]. Moreover, daily sleep disturbance was the most frequent problem reported by the participants. The main sources of construction worksite noise were included diesel power generators, cutting and welding processes, heavy machinery (e.g., trucks), and the transport of materials. In addition, most of the residents had heard the construction noise and believed that loudness to be the main reason for their annoyance. The analysis revealed that the measured mean noise level near the participants homes was 74.6 decibels (dB) (standard deviation [SD]: 7.1 dB] during the operation of construction machinery; in particular, it was 97 dB (SD: 2.2 dB) when the diesel generators were the primary source of the noise. In a Chinese study on local construction noise in residential buildings, noise levels between 67.8–73.9 dB were measured during nighttime construction activity [31]. The World Health Organization summarized that when night noise level exceeds 55 dB, adverse health effects occur frequently, a sizable proportion of the population is highly annoyed and sleep-disturbed, and there is evidence that the risk of CVD increases [32]. In addition, a recent meta-analysis indicated that the relative risk for IHD was significantly increased following transportation noise exposure, with the linear exposure-response starting at 50 dB [9].

On the other hand, due to the different acoustic characteristics for a diverse number of noise sources (e.g., sound level, frequency spectrum, time course, sound level rise time, and psychoacoustic measures), noise levels generated from different sources cannot be merged into one decibel indicator. In addition, various exposure-response curves related to CVDs have been generated for exposure to road traffic and aircraft noise [33]. In these curves, the relative risk of CVDs were highly variable, even at the same noise level. Regarding neighborhood noise, the association with CVDs or related symptoms has yet to be investigated. In addition, our study suggested significant associations between neighborhood noise and two symptoms related to stroke. Recently, one cross-sectional questionnaire study also suggested associations between neighborhood noise (e.g., equipment in the house, restaurants, cultural facilities, and commercial activities) and the incidence of stroke [34]. Therefore, an investigation of the exposure-response relationships between construction noise, neighborhood noise, and CVDs or related symptoms is required. The guidelines for environmental noise should likely be established on the grounds of the exposure-response relationships of respective noise sources.

There are several limitations associated with our study. First, we used a nationwide cross-sectional survey to examine the associations between perceived environmental noises and the subjective symptoms related to CVDs that the participants experienced within the past year. This cross-sectional design makes it difficult to determine the cause and effect of temporal relationships.

Second, this study was based on a self-reported questionnaire survey. Environmental reports from participants are highly subjective and the resulting inaccuracies may have resulted in bias. This is also true of the subjective, self-reported health outcome assessments used in this study, despite the use of a well-known questionnaire that has been validated in Japan.

Third, the limitation of this web-enabled methodology, as with other survey techniques, is the potential selection bias that exists if some individuals do not participate in the web panel because of concerns regarding the technology or other personal reasons. However, the conclusions drawn from this study may be unaffected by this limitation because the demographic characteristics and prevalence of CVDs between the study participants and other population-based statistical data using traditional measures showed similar tendencies.

Fourth, noise-induced hearing loss or presbycusis might be associated with effect of noise nuisance or annoyance; however, those were not investigated in this study. Occupational stress, educational level, passive smoking, and cholesterol level [7] were also not investigated. The geographic classifications (rural, suburban, or urban) of participants might be associated with CVDs. Thus, we investigated the proximity of housing to the main road and the visibility of the main road, and controlled for these in the models. In addition, we analyzed the associations between participant prefectures and CVDs and the related symptoms; however, the results were not significant.

Fifth, this study did not consider other environmental factors that may be associated with CVDs, such as exposure to fine particulate air pollution [35]. Environmental factors could potentially impact the effect estimates in the present study; however, long-term exposure to fine particulate matter and nocturnal traffic noise were both independently associated with subclinical atherosclerosis and may contribute to the association between road traffic and coronary artery disease [29]. In addition, a systematic review concluded that the confounding cardiovascular effects induced by noise or air pollutants was low [9,36], although this may be related to the quality of the methods used to assess air pollutants. Studies of the combined effects of noise and air pollution have revealed largely independent effects, which could be explained by different mechanisms pertaining to how both exposures impact health (cognitive and autonomic stress response vs. inflammatory processes) [33]. With regards to other environmental factors, one study reported that exposure to violent crimes, environmental noise, and proximity to a major road were independently associated with increased odds of CVDs [37]. Thus, these relevant factors may not substantially impact the observed associations in our study.

Finally, we analyzed mass variable factors that could introduce a systematic statistical bias. Therefore, we performed correlation analyses, univariate analyses, and three multivariable models, for which the results were similar for the different models used; thus, it is unlikely that the results from the analysis of risk factors on environmental noise annoyance were affected by a particular statistical model or the large number of statistical tests performed.

In conclusion, our findings suggest that the associations with CVD-related symptoms might be greater for construction, neighborhood, and automobile noise than that with railway and aircraft noise. While several previous studies focused specifically on transportation noise, our findings suggest that although transportation noise is an important risk factor for CVDs, construction and neighborhood noise may be of equal or greater importance. Moreover, annoyance caused by environmental noise appears to exert its effects in the form of CVD-related symptoms. Therefore, clarification of the relationships between the acoustic characteristics associated with different noise sources and CVDs or related symptoms is required in future studies.

## Supporting information

S1 Table Baseline characteristics by noise exposure status (*n* = 6181). (DOCX)

S2 Table Correlation between sources of environmental noise annoyance. (DOCX)

## References

[1] Lozano R, Naghavi M, Foreman K, Lim S, Shibuya K, Aboyans V, et al Global and regional mortality from 235 causes of death for 20 age groups in 1990 and 2010: a systematic analysis for the Global Burden of Disease Study 2010. Lancet. 2012;380:2095–2128. doi: 10.1016/S0140-6736(12)61728-0 23245604 10.1016/S0140-6736(12)61728-0 PMC10790329

[2] McAloon CJ, Boylan LM, Hamborg T, Stallard N, Osman F, Lim PB, et al The changing face of cardiovascular disease 2000–2012: An analysis of the world health organisation global health estimates data. Int J Cardiol. 2016;224:256–264. doi: 10.1016/j.ijcard.2016.09.026 27664572 10.1016/j.ijcard.2016.09.026

[3] Ishii S, Ogawa S, Akishita M. The state of health in older adults in Japan: trends in disability, chronic medical conditions and mortality. PLoS ONE. 2015;10:e0139639 doi: 10.1371/journal.pone.0139639 26431468 10.1371/journal.pone.0139639 PMC4592221

[4] Diez Roux AV. Residential environments and cardiovascular risk. J Urban Health. 2003:80(4):569–589. doi: 10.1093/jurban/jtg065 14709706 10.1093/jurban/jtg065 PMC3456219

[5] Münzel T, Gori T, Babisch W, Basner M. Cardiovascular effects of environmental noise exposure. Eur Heart J. 2014;35:829–836. doi: 10.1093/eurheartj/ehu030 24616334 10.1093/eurheartj/ehu030 PMC3971384

[6] Stansfeld S, Haines M, Brown B. Noise and health in the urban environment. Rev Environ Health. 2000;15:43–82. 10939085 10.1515/reveh.2000.15.1-2.43

[7] van Kempen EE, Kruize H, Boshuizen HC, Ameling CB, Staatsen BA, de Hollander AE. The association between noise exposure and blood pressure and ischemic heart disease: a meta-analysis. Environ Health Perspect. 2002;110:307–317. 11882483 10.1289/ehp.02110307 PMC1240772

[8] Gan WQ, Davies HW, Demers PA. Exposure to occupational noise and cardiovascular disease in the United States: the National Health and Nutrition Examination Survey 1999–2004. Occup Environ Med. 2011;68:183–190. doi: 10.1136/oem.2010.055269 20924023 10.1136/oem.2010.055269

[9] Vienneau D, Schindler C, Perez L. The relationship between transportation noise exposure and ischemic heart disease: a meta-analysis. Environ Res. 2015;138:372–380. doi: 10.1016/j.envres.2015.02.023 25769126 10.1016/j.envres.2015.02.023

[10] Pyko A, Eriksson C, Oftedal B, Hilding A, Östenson CG, Krog NH, et al Exposure to traffic noise and markers of obesity. Occup Environ Med. 2015;72:594–601. doi: 10.1136/oemed-2014-102516 26009579 10.1136/oemed-2014-102516

[11] Héritier H, Vienneau D, Foraster M, Eze IC, Schaffner E, Thiesse L, et al Transportation noise exposure and cardiovascular mortality: a nationwide cohort study from Switzerland. Eur J Epidemiol. 2017;32:307–315. doi: 10.1007/s10654-017-0234-2 28280950 10.1007/s10654-017-0234-2

[12] Golmohammadi R, Mohammadi H, Bayat H, Habibi Mohraz M, Soltanian AR. Noise annoyance due to construction worksites. J Res Health Sci. 2013;13:201–207. 24077480

[13] Andrews EB, Eaton SC, Hollis KA, Hopkins JS, Ameen V, Hamm LR, et al Prevalence and demographics of irritable bowel syndrome: results from a large web-based survey. Aliment Pharmacol Ther. 2005;22:935–942. doi: 10.1111/j.1365-2036.2005.02671.x 16268967 10.1111/j.1365-2036.2005.02671.x

[14] Johannes CB, Le TK, Zhou X, Johnston JA, Dworkin RH. The prevalence of chronic pain in United States adults: results of an Internet-based survey. J Pain. 2010;11:1230–1239. doi: 10.1016/j.jpain.2010.07.002 20797916 10.1016/j.jpain.2010.07.002

[15] Krogsgaard LR, Engsbro AL, Bytzer P. The epidemiology of irritable bowel syndrome in Denmark. A population-based survey in adults ≤50 years of age. Scand J Gastroenterol. 2013;48:523–529. doi: 10.3109/00365521.2013.775328 23506174 10.3109/00365521.2013.775328

[16] Reimer C, Bytzer P. A population-based survey to assess troublesome symptoms in gastroesophageal reflux disease. Scand J Gastroenterol. 2009;44: 394–400. doi: 10.1080/00365520802600987 19065448 10.1080/00365520802600987

[17] Azuma K, Uchiyama I, Katoh T, Ogata H, Arashidani K, Kunugita N. Prevalence and Characteristics of Chemical Intolerance: A Japanese Population-Based Study. Arch Environ Occup Health. 2015;70:341–353. doi: 10.1080/19338244.2014.926855 25137616 10.1080/19338244.2014.926855

[18] Ito M, Bentley KH, Oe Y, Nakajima S, Fujisato H, Kato N, et al Assessing Depression Related Severity and Functional Impairment: The Overall Depression Severity and Impairment Scale (ODSIS). PLoS ONE. 2015;10: e0122969 doi: 10.1371/journal.pone.0122969 25874558 10.1371/journal.pone.0122969 PMC4395441

[19] Sasaki M, Yoshida K, Adachi Y, Furukawa M, Itazawa T, Odajima H. Environmental factors associated with childhood eczema: Findings from a national web-based survey. Allergol Int. 2016;65:420–424. doi: 10.1016/j.alit.2016.03.007 27134054 10.1016/j.alit.2016.03.007

[20] Macromill. Lifestyle Survey [in Japanese]. Tokyo, Japan: Macromill Netresearch Institute, Macromil; 2013.

[21] Ministry of Internal Affairs and Communications (MIAC). 2010 Population Census. Tokyo, Japan: Statistics Bureau, MIAC; 2011.

[22] Konishi M, Kondou H, Okada K. Health status, life habits, and social background among the JPHC study participants at baseline survey. Japan Public Health Center-based Prospective Study on Cancer and Cardiovascular Diseases. J Epidemiol. 2001;11(6 Suppl):S57–74. 11763140 10.2188/jea.11.6sup_57 PMC11858364

[23] Fields JM, De Jong RG, Gjestland T, Flindell IH, Job RFS, Kurra S, et al Standardized general-purpose noise reaction questions for community noise surveys: research and a recommendation. J Sound Vib. 2001;242:641–679.

[24] ISO/TC43/WG. Assessment of noise annoyance by means of social and socio-acoustic surveys. ISO; 2003.

[25] Dormann CF, Elith J, Bacher S, Buchmann C, Carl G, Carré G, et al Collinearity: a review of methods to deal with it and a simulation study evaluating their performance. Ecography. 2013;36:27–46.

[26] Nafiu OO, Onyewuche V. Association of abdominal obesity in children with perioperative respiratory adverse events. J Perianesth Nurs. 2014;29:84–93. doi: 10.1016/j.jopan.2013.03.010 24661478 10.1016/j.jopan.2013.03.010

[27] Bareinboim E, Pearl J. Causal inference and the data-fusion problem. Proc Natl Acad Sci USA. 2016;113:7345–7352. doi: 10.1073/pnas.1510507113 27382148 10.1073/pnas.1510507113 PMC4941504

[28] Ministry of Health, Labour and Welfare (MHLW). Summary Report of Comprehensive Survey of Living Conditions 2013 [in Japanese]. Tokyo, Japan: MHLW; 2014.

[29] Kälsch H, Henning F, Moebus S, Möhlenkamp S, Dragano N, Jakobs H, et al Are air pollution and traffic noise independently associated with atherosclerosis–the Heinz Nixdorf Recall Study. Eur Heart J. 2014;35:853–860. doi: 10.1093/eurheartj/eht426 24194529 10.1093/eurheartj/eht426

[30] WHO Europe. Burden of disease from environmental noise: quantification of healthy life years lost in Europe. World Health Organization Regional Office for Europe, Copenhagen, 2011.

[31] Xiao J, Li X, Zhang Z. DALY-Based Health Risk Assessment of Construction Noise in Beijing, China. Int J Environ Res Public Health. 2016;13:1045 doi: 10.3390/ijerph13111045 27792207 10.3390/ijerph13111045 PMC5129255

[32] WHO Europe. Night noise guidelines for Europe. World Health Organization Regional Office for Europe, Copenhagen, 2009.

[33] Basner M, Babisch W, Davis A, Brink M, Clark C, Janssen S, et al Auditory and non-auditory effects of noise on health. Lancet. 2014;383:1325–1332. doi: 10.1016/S0140-6736(13)61613-X 24183105 10.1016/S0140-6736(13)61613-X PMC3988259

[34] Vandasova Z, Vencálek O, Puklová V. Specific and combined subjective responses to noise and their association with cardiovascular diseases. Noise Health. 2016;18:338–346. doi: 10.4103/1463-1741.195800 27991465 10.4103/1463-1741.195800 PMC5227014

[35] Brook RD, Rajagopalan S, Pope CA III, Brook JR, Bhatnagar A, Diez-Roux AV, et al Particulate matter air pollution and cardiovascular disease: An update to the scientific statement from the American Heart Association. Circulation. 2010;121:2331–2378. doi: 10.1161/CIR.0b013e3181dbece1 20458016 10.1161/CIR.0b013e3181dbece1

[36] Tétreault LF, Perron S, Smargiassi A. Cardiovascular health, traffic-related air pollution and noise: are associations mutually confounded? A systematic review. Int J Public Health. 2013;58:649–666. doi: 10.1007/s00038-013-0489-7 23887610 10.1007/s00038-013-0489-7 PMC3907786

[37] Chum A, O'Campo P. Cross-sectional associations between residential environmental exposures and cardiovascular diseases. BMC Public Health. 2015;15:438 doi: 10.1186/s12889-015-1788-0 25924669 10.1186/s12889-015-1788-0 PMC4438471

