# Supplementary material for: Association between environmental noise and subjective symptoms related to cardiovascular diseases among elderly individuals in Japan
Source: PLoS One. 2017 Nov 16;12(11):e0188236. doi: 10.1371/journal.pone.0188236 (PMC5690640; doi:10.1371/journal.pone.0188236)
Supplement: S1 Table — (DOCX) [file pone.0188236.s001.docx]

Online supporting information for the following article published in *PLoS ONE*

**Association between environmental noise and subjective symptoms related to cardiovascular diseases among elderly individuals in Japan**

Kenichi Azuma^1,2^, KIwao Uchiyama^2^

*1 Corresponding Author: Department of Environmental Medicine and Behavioral Science, Kindai University Faculty of Medicine, Osakasayama, Osaka, Japan*

*2 Sick-house Medical Science Laboratory, Division of Basic Research, Louis Pasteur Center for Medical Research, Kyoto, Japan*

**Table S1-a.** Baseline characteristics by noise exposure status (*n* = 6181).

| Potential confounder | Awakening during the night due to noise | | *P* value ^b^ |
| --- | --- | --- | --- |
|  | with | without |  |
| Age, *n* (%) |  |  |  |
| <75 | 1367 (83.9) | 4020 (86.5) | 0.004 |
| ≥75 | 164 (10.7) | 630 (13.5) |  |
| Male, *n* (%) | 651 (42.5) | 2367 (50.9) | <0.001 |
| Employment, *n* (%) |  |  |  |
| Employed | 131 (8.6) | 335 (7.2) | <0.001 |
| Self-employed | 125 (8.2) | 410 (8.8) |  |
| Housewife/househusband | 566 (37.0) | 1477 (31.8) |  |
| Part-time employed | 136 (8.9) | 360 (7.7) |  |
| Unemployed | 450 (29.4) | 1573 (33.8) |  |
| Others | 123 (8.0) | 495 (10.6) |  |
| Degree of obesity, *n* (%) ^a^ |  |  |  |
| Underweight | 129 (8.4) | 305 (6.6) | 0.019 |
| Normal | 1128 (73.7) | 3426 (73.7) |  |
| Obesity (class I) | 249 (16.3) | 833 (17.9) |  |
| Obesity (class II) | 20 (1.3) | 76 (1.6) |  |
| Obesity (class III) | 5 (0.3) | 4 (0.1) |  |
| Obesity (class IV) | 0 (0.0) | 2 (0.0) |  |
| Medical history of parents (with), *n* (%) |  |  |  |
| Stroke  IHDs | 326 (21.3)  202 (13.2) | 996 (21.4)  522 (11.2) | 0.917  0.038 |
| Smoking status, *n* (%) |  |  |  |
| Current/every day | 139 (9.1) | 513 (11.0) | 0.121 |
| Current/sometimes | 17 (1.1) | 44 (0.9) |  |
| Former | 476 (31.1) | 1475 (31.7) |  |
| Never | 899 (58.7) | 2618 (56.3) |  |
| Drinking habits, *n* (%) |  |  |  |
| Once or more a week | 731 (47.7) | 2231 (48.0) | 0.875 |
| Less than once a week or never | 800 (52.3) | 2419 (52.0) |  |
| Regular exercise, *n* (%) |  |  |  |
| Current/always  Current/sometimes  Former  Never | 525 (34.3  649 (42.4)  221 (14.4)  136 (8.9) | 1704 (36.6)  1938 (41.7)  534 (11.5)  474 (10.2) | 0.007 |
| Sufficiency of sleep duration, *n* (%) |  |  |  |
| Short  Average  Good  Long | 566 (37.0)  160 (10.5)  764 (49.9)  41 (2.7) | 1044 (22.5)  440 (9.5)  3059 (65.8)  107 (2.3) | <0.001 |
| Depth of sleep, *n* (%) |  |  |  |
| Deep  Fairly deep  Average  Fairly light  Light | 93 (6.1)  686 (44.8)  43 (2.8)  557 (36.4)  152 (9.9) | 1027 (22.1)  2653 (57.1)  117 (2.5)  709 (15.2)  144 (3.1) | <0.001 |
| Proximity of housing to the main road, *n* (%) |  |  |  |
| Along with road  ≤50 m  50 to ≤150 m  150 to ≤500 m  >500 m | 198 (12.9)  281 (18.4)  294 (19.2)  414 (27.0)  344 (22.5) | 578 (12.4)  816 (17.5)  904 (19.4)  1290 (27.7)  1062 (22.8) | 0.916 |
| Visibility of the main road, *n* (%) |  |  |  |
| Good  Partly  Poor | 189 (12.3)  394 (25.7)  948 (61.9) | 522 (11.2)  1118 (24.0)  3010 (64.7) | 0.134 |
| Bedroom is close to main road (with), *n* (%) | 207 (13.5) | 632 (13.6) | 0.944 |
| Duration of residence, *n* (%) |  |  |  |
| <3 years  3 to <20 years  ≥ 20 years | 86 (5.6)  503 (32.9)  942 (61.5) | 191 (4.1)  1428 (30.7)  3031 (65.2) | 0.007 |
| Housing structure, *n* (%) |  |  |  |
| Wooden  Reinforced concrete  Others | 932 (60.9)  512 (33.4)  87 (5.7) | 2808 (60.4)  1580 (34.0)  262 (5.6) | 0.929 |
| Use of an unvented-heating appliance in winter (with), *n* (%) | 724 (47.3) | 2129 (45.8) | 0.306 |
| Use of incense in the house (with), *n* (%) | 816 (53.3) | 2490 (53.5) | 0.865 |

^a^ Data were missing for four participants. Abbreviations: IHD, ischemic heart diseases. ^b^ *P* value from χ^2^ test.

**Table S1-b.** Baseline characteristics by noise exposure status (*n* = 6181).

| Potential confounder | Daytime automobile noise annoyance ^b^ | | | | | | *P* value ^c^ |
| --- | --- | --- | --- | --- | --- | --- | --- |
|  | (1) | (2) | (3) | (4) | (5) | (6) |  |
| Age, *n* (%) |  |  |  |  |  |  |  |
| <75 | 1194 (83.9) | 1570 (89.0) | 2237 (87.2) | 324 (90.5) | 40 (87.0) | 22 (84.6) | <0.001 |
| ≥75 | 229 (16.1) | 194 (11.0) | 327 (12.8) | 34 (9.5) | 6 (13.0) | 4 (15.1) |  |
| Male, *n* (%) | 750 (52.7) | 870 (49.3) | 1188 (46.3) | 174 (48.6) | 22 (47.8) | 14 (53.8) | 0.009 |
| Employment, *n* (%) |  |  |  |  |  |  |  |
| Employed | 117 (8.2) | 130 (7.4) | 191 (7.4) | 24 (6.7) | 3 (6.5) | 1 (3.8) | 0.110 |
| Self-employed | 109 (7.7) | 149 (8.4) | 246 (9.6) | 30 (8.4) | 1 (2.2) | 0 (0.0) |  |
| Housewife/househusband | 432 (30.4) | 583 (33.0) | 888 (34.6) | 117 (32.7) | 14 (30.4) | 9 (34.6) |  |
| Part-time employed | 108 (7.6) | 139 (7.9) | 209 (8.2) | 30 (8.4) | 7 (15.2) | 3 (11.5) |  |
| Unemployed | 487 (34.2) | 602 (34.1) | 788 (30.7) | 118 (33.0) | 17 (37.0) | 11 (42.3) |  |
| Others | 170 (11.9) | 161 (9.1) | 242 (9.4) | 39 (10.9) | 4 (8.7) | 2 (7.7) |  |
| Degree of obesity, *n* (%) ^a^ |  |  |  |  |  |  |  |
| Underweight | 94 (6.6) | 116 (6.6) | 193 (7.5) | 26 (7.3) | 3 (6.5) | 2 (7.7) | 0.660 |
| Normal | 1050 (73.8) | 1288 (73.1) | 1882 (73.5) | 279 (77.9) | 37 (80.4) | 18 (69.2) |  |
| Obesity (class I) | 255 (17.9) | 332 (18.8) | 439 (17.1) | 46 (12.8) | 4 (8.7) | 6 (23.1) |  |
| Obesity (class II) | 22 (1.5) | 25 (1.4) | 42 (1.6) | 5 (1.4) | 2 (4.3) | 0 (0.0) |  |
| Obesity (class III) | 2 (0.1) | 1 (0.1) | 4 (0.2) | 2 (0.6) | 0 (0.0) | 0 (0.0) |  |
| Obesity (class IV) | 0 (0.0) | 1 (0.1) | 1 (0.0) | 0 (0.0) | 0 (0.0) | 0 (0.0) |  |
| Medical history of parents (with), *n* (%) |  |  |  |  |  |  |  |
| Stroke  IHDs | 323 (22.7)  166 (11.7) | 400 (22.7)  191 (10.8) | 518 (20.2)  303 (11.8) | 69 (19.3)  52 (14.5) | 8 (17.4)  9 (19.6) | 4 (15.4)  3 (11.5) | 0.201  0.232 |
| Smoking status, *n* (%) |  |  |  |  |  |  |  |
| Current/every day | 145 (10.2) | 199 (11.3) | 270 (10.5) | 30 (8.4) | 2 (4.3) | 6 (23.1) | 0.023 |
| Current/sometimes | 22 (1.5) | 13 (0.7) | 22 (0.9) | 3 (0.8) | 1 (2.2) | 0 (0.0) |  |
| Former | 464 (32.6) | 586 (33.2) | 759 (29.6) | 120 (33.5) | 18 (39.1) | 4 (15.4) |  |
| Never | 792 (55.7) | 966 (54.8) | 1513 (59.0) | 205 (57.3) | 25 (54.3) | 16 (61.5) |  |
| Drinking habits, *n* (%) |  |  |  |  |  |  |  |
| Once or more a week | 658 (46.2) | 872 (49.4) | 1204 (47.0) | 188 (52.5) | 24 (52.2) | 16 (61.5) | 0.092 |
| Less than once a week or never | 765 (53.8) | 892 (50.6) | 1360 (53.0) | 170 (47.5) | 22 (47.8) | 10 (38.5) |  |
| Regular exercise, *n* (%) |  |  |  |  |  |  |  |
| Current/always  Current/sometimes  Former  Never | 535 (37.6)  565 (39.7)  170 (11.9)  153 (10.8) | 666 (37.8)  727 (41.2)  218 (12.4)  153 (8.7) | 867 (33.8)  1121 (43.7)  317 (12.4)  259 (10.1) | 134 (37.4)  149 (41.6)  42 (11.7)  33 (9.2) | 21 (45.7)  13 (28.3)  4 (8.7)  8 (17.4) | 6 (23.1)  12 (46.2)  4 (15.4)  4 (15.4) | 0.087 |
| Sufficiency of sleep duration, *n* (%) |  |  |  |  |  |  |  |
| Short  Average  Good  Long | 330 (23.2)  140 (9.8)  915 (64.3)  38 (2.7) | 423 (24.0)  156 (8.8)  1153 (65.4)  32 (1.8) | 705 (27.5)  265 (10.3)  1533 (59.8)  61 (2.4) | 124 (34.6)  31 (8.7)  195 (54.5)  8 (2.2) | 17 (37.0)  7 (15.2)  16 (34.8)  6 (13.0) | 11 (42.3)  1 (3.8)  11 (42.3)  3 (11.5) | <0.001 |
| Depth of sleep, *n* (%) |  |  |  |  |  |  |  |
| Deep  Fairly deep  Average  Fairly light  Light | 348 (24.5)  747 (52.5)  39 (2.7)  244 (17.1)  45 (3.2) | 338 (19.2)  991 (56.2)  35 (2.0)  334 (18.9)  66 (3.7) | 389 (15.2)  1391 (54.3)  73 (2.8)  568 (22.2)  143 (5.6) | 38 (10.6)  179 (50.0)  11 (3.1)  103 (28.8)  27 (7.5) | 2 (4.3)  19 (41.3)  1 (2.2)  12 (26.1)  12 (26.1) | 5 (19.2)  12 (46.2)  1 (3.8)  5 (19.2)  3 (11.5) | <0.001 |
| Proximity of housing to the main road, *n* (%) |  |  |  |  |  |  |  |
| Along with road  ≤50 m  50 to ≤150 m  150 to ≤500 m  >500 m | 73 (5.1)  202 (14.2)  274 (19.3)  455 (32.0)  419 (29.4) | 198 (11.2)  335 (19.0)  354 (20.1)  477 (27.0)  400 (22.7) | 389 (15.2)  503 (19.6)  505 (19.7)  672 (26.2)  495 (19.3) | 93 (26.0)  47 (13.1)  56 (15.6)  85 (23.7)  77 (21.5) | 16 (34.8)  7 (15.2)  9 (19.6)  8 (17.4)  6 (13.0) | 7 (26.9)  3 (11.5)  0 (0.0)  7 (26.9)  9 (34.6) | <0.001 |
| Visibility of the main road, *n* (%) |  |  |  |  |  |  |  |
| Good  Partly  Poor | 85 (6.0)  245 (17.2)  1093 (76.8) | 167 (9.5)  422 (23.9)  1175 (66.6) | 364 (14.2)  724 (28.2)  1476 (57.6) | 76 (21.2)  103 (28.8)  179 (50.0) | 14 (30.4)  11 (23.9)  21 (45.7) | 5 (19.2)  7 (26.9)  14 (53.8) | <0.001 |
| Bedroom is close to main road (with), *n* (%) | 117 (8.2) | 226 (12.8) | 407 (15.9) | 70 (19.6) | 14 (30.4) | 5 (19.2) | <0.001 |
| Duration of residence, *n* (%) |  |  |  |  |  |  |  |
| <3 years  3 to <20 years  ≥ 20 years | 89 (6.3)  448 (31.5)  886 (62.3) | 75 (4.3)  537 (30.4)  1152 (65.3) | 102 (4.0)  799 (31.2)  1663 (64.9) | 10 (2.8)  123 (34.4)  225 (62.8) | 0 (0.0)  14 (30.4)  32 (69.6) | 1 (3.8)  10 (38.5)  15 (57.7) | 0.032 |
| Housing structure, *n* (%) |  |  |  |  |  |  |  |
| Wooden  Reinforced concrete  Others | 889 (62.5)  454 (31.9)  80 (5.6) | 1068 (60.5)  596 (33.8)  100 (5.7) | 1517 (59.2)  901 (35.1)  146 (5.7) | 219 (61.2)  120 (33.5)  19 (5.3) | 28 (60.9)  15 (32.6)  3 (6.5) | 19 (73.1)  6 (23.1)  1 (3.8) | 0.776 |
| Use of an unvented-heating appliance in winter (with), *n* (%) | 647 (45.5) | 842 (47.7) | 1169 (45.6) | 153 (42.7) | 30 (65.2) | 12 (46.2) | 0.056 |
| Use of incense in the house (with), *n* (%) | 704 (49.5) | 920 (52.2) | 1441 (56.2) | 196 (54.7) | 30 (65.2) | 15 (57.7) | <0.001 |

^a^ Data were missing for four participants. ^b^ The six levels were (1) no noise, (2) not annoyed, (3) less annoyed, (4) moderately annoyed, (5) annoyed, and (6) highly annoyed. Abbreviations: IHD, ischemic heart diseases. ^c^ *P* value from χ^2^ test.

**Table S1-c.** Baseline characteristics by noise exposure status (*n* = 6181).

| Potential confounder | Nocturnal automobile noise annoyance ^b^ | | | | | | *P* value ^c^ |
| --- | --- | --- | --- | --- | --- | --- | --- |
|  | (1) | (2) | (3) | (4) | (5) | (6) |  |
| Age, *n* (%) |  |  |  |  |  |  |  |
| <75 | 1148 (84.2) | 1442 (89.6) | 2225 (87.0) | 468 (88.8) | 77 (83.7) | 27 (87.1) | <0.001 |
| ≥75 | 216 (15.8) | 167 (10.4) | 333 (13.0) | 59 (11.2) | 15 (16.3) | 4 (12.9) |  |
| Male, *n* (%) | 713 (52.3) | 772 (48.0) | 1201 (47.0) | 271 (51.4) | 43 (46.7) | 18 (58.1) | 0.022 |
| Employment, *n* (%) |  |  |  |  |  |  |  |
| Employed | 105 (7.7) | 118 (7.3) | 189 (7.4) | 41 (7.8) | 12 (13.0) | 1 (3.2) | 0.054 |
| Self-employed | 105 (7.7) | 140(8.7) | 240 (9.4) | 44 (8.3) | 5 (5.4) | 1 (3.2) |  |
| Housewife/househusband | 414 (30.4) | 540 (33.6) | 892 (34.9) | 158 (30.0) | 30 (32.6) | 9 (29.0) |  |
| Part-time employed | 107 (7.8) | 128 (8.0) | 202 (7.9) | 47 (8.9) | 9 (9.8) | 3 (9.7) |  |
| Unemployed | 465 (34.1) | 544 (33.8) | 788 (30.8) | 187 (35.5) | 25 (27.2) | 14 (45.2) |  |
| Others | 168 (12.3) | 139 (8.6) | 247 (9.7) | 50 (9.5) | 11 (12.0) | 3 (9.7) |  |
| Degree of obesity, *n* (%) ^a^ |  |  |  |  |  |  |  |
| Underweight | 93 (6.8) | 111 (6.9) | 178 (7.0) | 45 (8.6) | 7 (7.6) | 0 (0.0) | 0.146 |
| Normal | 1000 (73.3) | 1177 (73.2) | 1878 (73.5) | 407 (77.4) | 67 (72.8) | 25 (80.6) |  |
| Obesity (class I) | 247 (18.1) | 298 (18.5) | 453 (17.7) | 64 (12.2) | 14 (15.2) | 6 (19.4) |  |
| Obesity (class II) | 22 (1.6) | 20 (1.2) | 42 (1.6) | 9 (1.7) | 3 (3.3) | 0 (0.0) |  |
| Obesity (class III) | 2 (0.1) | 1 (0.1) | 5 (0.2) | 0 (0.0) | 1 (1.1) | 0 (0.0) |  |
| Obesity (class IV) | 1 (0.0) | 1 (0.1) | 0 (0.0) | 1 (0.2) | 0 (0.0) | 0 (0.0) |  |
| Medical history of parents (with), *n* (%) |  |  |  |  |  |  |  |
| Stroke  IHDs | 304 (22.3)  161 (11.8) | 364 (22.6)  174 (10.8) | 514 (20.1)  296 (11.6) | 113 (21.4)  72 (13.7) | 19 (20.7)  17 (18.5) | 8 (25.8)  4 (12.9) | 0.409  0.195 |
| Smoking status, *n* (%) |  |  |  |  |  |  |  |
| Current/every day | 131 (9.6) | 177 (11.0) | 277 (10.8) | 58 (11.0) | 5 (5.4) | 4 (12.9) | 0.482 |
| Current/sometimes | 16 (1.2) | 17 (1.1) | 20 (0.8) | 8 (1.5) | 0 (0.0) | 0 (0.0) |  |
| Former | 455 (33.4) | 505 (31.4) | 777 (30.4) | 174 (33.0) | 33 (35.9) | 7 (22.6) |  |
| Never | 762 (55.9) | 910 (56.6) | 1484 (58.0) | 287 (54.5) | 54 (58.7) | 20 (64.5) |  |
| Drinking habits, *n* (%) |  |  |  |  |  |  |  |
| Once or more a week | 627 (46.0) | 805 (50.0) | 1210 (47.3) | 254 (48.2) | 47 (51.1) | 19 (61.3) | 0.159 |
| Less than once a week or never | 737 (54.0) | 804 (50.0) | 1348 (52.7) | 273 (51.8) | 45 (48.9) | 12 (38.7) |  |
| Regular exercise, *n* (%) |  |  |  |  |  |  |  |
| Current/always  Current/sometimes  Former  Never | 508 (37.2)  544 (39.9)  162 (11.9)  150 (11.0) | 608 (37.8)  667 (41.5)  194 (12.1)  140 (8.7) | 884 (34.6)  1113 (43.5)  318 (12.4)  243 (9.5) | 186 (35.3)  217 (41.2)  67 (12.7)  57 (10.8) | 35 (38.0)  34 (37.0)  9 (9.8)  14 (15.2) | 8 (25.8)  12 (38.7)  5 (16.1)  6 (19.4) | 0.183 |
| Sufficiency of sleep duration, *n* (%) |  |  |  |  |  |  |  |
| Short  Average  Good  Long | 321 (23.5)  133 (9.8)  875 (64.1)  35 (2.6) | 408 (25.4)  130 (8.1)  1038 (64.5)  33 (2.1) | 647 (25.3)  263 (10.3)  1590 (62.2)  58 (2.3) | 193 (36.6)  60 (11.4)  262 (49.7)  12 (2.3) | 31 (33.7)  12 (13.0)  42 (45.7)  7 (7.6) | 10 (32.3)  2 (6.5)  16 (51.6)  3 (9.7) | <0.001 |
| Depth of sleep, *n* (%) |  |  |  |  |  |  |  |
| Deep  Fairly deep  Average  Fairly light  Light | 337 (24.7)  710 (52.1)  36 (2.6)  233 (17.1)  48 (3.5) | 319 (19.8)  909 (56.5)  32 (2.0)  292 (18.1)  57 (3.5) | 398 (15.6)  1417 (55.4)  68 (2.7)  545 (21.3)  130 (5.1) | 52 (9.9)  246 (46.7)  21 (4.0)  165 (31.3)  43 (8.2) | 10 (10.9)  43 (46.7)  2 (2.2)  25 (27.2)  12 (13.0) | 4 (12.9)  14 (45.2)  1 (3.2)  6 (19.4)  6 (19.4) | <0.001 |
| Proximity of housing to the main road, *n* (%) |  |  |  |  |  |  |  |
| Along with road  ≤50 m  50 to ≤150 m  150 to ≤500 m  >500 m | 72 (5.3)  189 (13.9)  260 (19.1)  438 (32.1)  405 (29.7) | 185 (11.5)  294 (18.3)  331 (20.6)  428 (26.6)  371 (23.1) | 366 (14.3)  485 (19.0)  508 (19.9)  689 (26.9)  510 (19.9) | 113 (21.4)  110 (20.9)  85 (16.1)  125 (23.7)  94 (17.8) | 27 (29.3)  15 (16.3)  13 (14.1)  19 (20.7)  18 (19.6) | 13 (41.9)  4 (12.9)  1 (3.2)  5 (16.1)  8 (25.8) | <0.001 |
| Visibility of the main road, *n* (%) |  |  |  |  |  |  |  |
| Good  Partly  Poor | 81 (5.9)  233 (17.1)  1050 (77.0) | 156 (9.7)  374 (23.2)  1079 (67.1) | 338 (13.2)  714 (27.9)  1506 (58.9) | 104 (19.7)  160 (30.4)  263 (49.9) | 22 (23.9)  23 (25.0)  47 (51.1) | 10 (32.3)  8 (25.8)  13 (41.9) | <0.001 |
| Bedroom is close to main road (with), *n* (%) | 120 (8.8) | 192 (11.9) | 397 (15.5) | 98 (18.6) | 22 (23.9) | 10 (32.3) | <0.001 |
| Duration of residence, *n* (%) |  |  |  |  |  |  |  |
| <3 years  3 to <20 years  ≥ 20 years | 87 (6.4)  433 (31.7)  844 (61.9) | 68 (4.2)  515 (32.0)  1026 (63.8) | 104 (4.1)  771 (30.1)  1683 (65.8) | 15 (2.8)  166 (31.5)  346 (65.4) | 2 (2.2)  34 (37.0)  56 (60.9) | 1 (3.2)  12 (38.7)  18 (58.1) | 0.014 |
| Housing structure, *n* (%) |  |  |  |  |  |  |  |
| Wooden  Reinforced concrete  Others | 854 (62.6)  434 (31.8)  76 (5.6) | 951 (59.1)  565 (35.1)  93 (5.8) | 1529 (59.8)  878 (34.3)  151 (5.9) | 332 (63.0)  173 (32.8)  22 (4.2) | 52 (56.5)  33 (35.9)  7 (7.6) | 22 (71.0)  9 (29.0)  0 (0.0) | 0.363 |
| Use of an unvented-heating appliance in winter (with), *n* (%) | 615 (45.1) | 759 (47.2) | 1171 (45.8) | 240 (45.5) | 49 (53.3) | 19 (61.3) | 0.283 |
| Use of incense in the house (with), *n* (%) | 677 (49.6) | 828 (51.5) | 1434 (56.1) | 289 (54.8) | 58 (63.0) | 20 (64.5) | <0.001 |

^a^ Data were missing for four participants. ^b^ The six levels were (1) no noise, (2) not annoyed, (3) less annoyed, (4) moderately annoyed, (5) annoyed, and (6) highly annoyed. Abbreviations: IHD, ischemic heart diseases. ^c^ *P* value from χ^2^ test.

**Table S1-d.** Baseline characteristics by noise exposure status (*n* = 6181).

| Potential confounder | Daytime neighborhood noise annoyance ^b^ | | | | | | *P* value ^c^ |
| --- | --- | --- | --- | --- | --- | --- | --- |
|  | (1) | (2) | (3) | (4) | (5) | (6) |  |
| Age, *n* (%) |  |  |  |  |  |  |  |
| <75 | 1586 (84.7) | 1836 (88.4) | 1648 (87.1) | 244 (93.5) | 53 (94.6) | 20 (87.0) | <0.001 |
| ≥75 | 287 (15.3) | 241 (11.6) | 243 (12.9) | 17 (6.5) | 3 (5.4) | 3 (13.0) |  |
| Male, *n* (%) | 982 (52.4) | 1036 (49.9) | 840 (44.4) | 123 (47.1) | 24 (42.9) | 13 (56.5) | <0.001 |
| Employment, *n* (%) |  |  |  |  |  |  |  |
| Employed | 144 (7.7) | 158 (7.6) | 131 (6.9) | 29 (11.1) | 2 (3.6) | 2 (8.7) | 0.051 |
| Self-employed | 169 (9.0) | 166 (8.0) | 172 (9.1) | 21 (8.0) | 5 (8.9) | 2 (8.7) |  |
| Housewife/househusband | 572 (30.5) | 705 (33.9) | 657 (34.7) | 86 (33.0) | 17 (30.4) | 6 (26.1) |  |
| Part-time employed | 130 (6.9) | 171 (8.2) | 159 (8.4) | 22 (8.4) | 10 (17.9) | 4 (17.4) |  |
| Unemployed | 644 (34.4) | 684 (32.9) | 593 (31.4) | 76 (29.1) | 18 (32.1) | 8 (34.8) |  |
| Others | 214 (11.4) | 193 (9.3) | 179 (9.5) | 27 (10.3) | 4 (7.1) | 1 (4.3) |  |
| Degree of obesity, *n* (%) ^a^ |  |  |  |  |  |  |  |
| Underweight | 130 (6.9) | 139 (6.7) | 147 (7.8) | 12 (4.6) | 5 (8.9) | 1 (4.3) | 0.692 |
| Normal | 1368 (73.0) | 1525 (73.5) | 1397 (74.0) | 204 (78.2) | 41 (73.2) | 19 (82.6) |  |
| Obesity (class I) | 341 (18.2) | 379 (18.3) | 306 (16.2) | 44 (16.9) | 10 (17.9) | 2 (8.7) |  |
| Obesity (class II) | 32 (1.7) | 29 (1.4) | 33 (1.7) | 1 (0.4) | 0 (0.0) | 1 (4.3) |  |
| Obesity (class III) | 2 (0.1) | 3 (0.1) | 4 (0.2) | 0 (0.0) | 0 (0.0) | 0 (0.0) |  |
| Obesity (class IV) | 0 (0.0) | 0 (0.0) | 2 (0.0) | 0 (0.0) | 0 (0.0) | 0 (0.0) |  |
| Medical history of parents (with), *n* (%) |  |  |  |  |  |  |  |
| Stroke  IHDs | 391 (20.9)  212 (11.3) | 467 (22.5)  244 (11.7) | 392 (20.7)  217 (11.5) | 60 (23.0)  43 (16.5) | 10 (17.9)  7 (12.5) | 2 (8.7)  1 (4.3) | 0.383  0.196 |
| Smoking status, *n* (%) |  |  |  |  |  |  |  |
| Current/every day | 197 (10.5) | 228 (11.0) | 194 (10.3) | 22 (8.4) | 8 (14.3) | 3 (13.0) | 0.041 |
| Current/sometimes | 20 (1.1) | 15 (0.7) | 23 (1.2) | 2 (0.8) | 0 (0.0) | 1 (4.3) |  |
| Former | 635 (33.9) | 672 (32.4) | 534 (28.2) | 83 (31.8) | 19 (33.9) | 8 (34.8) |  |
| Never | 1021 (54.5) | 1162 (55.9) | 1140 (60.3) | 154 (59.0) | 29 (51.8) | 11 (47.8) |  |
| Drinking habits, *n* (%) |  |  |  |  |  |  |  |
| Once or more a week | 876 (46.8) | 1016 (48.9) | 900 (47.6) | 131 (50.2) | 28 (50.0) | 11 (47.8) | 0.771 |
| Less than once a week or never | 997 (53.2) | 1061 (51.1) | 991 (52.4) | 130 (49.8) | 28 (50.0) | 12 (52.2) |  |
| Regular exercise, *n* (%) |  |  |  |  |  |  |  |
| Current/always  Current/sometimes  Former  Never | 689 (36.8)  749 (40.0)  237 (12.7)  198 (10.6) | 756 (36.4)  869 (41.8)  256 (12.3)  196 (9.4) | 661 (35.0)  826 (43.7)  228 (12.1)  176 (9.3) | 90 (34.5)  109 (41.8)  29 (11.1)  33 (12.6) | 25 (44.6)  23 (41.1)  4 (7.1)  4 (7.1) | 8 (34.8)  11 (47.8)  1 (4.3)  3 (13.0) | 0.589 |
| Sufficiency of sleep duration, *n* (%) |  |  |  |  |  |  |  |
| Short  Average  Good  Long | 457 (24.4)  176 (9.4)  1193 (63.7)  47 (2.5) | 505 (24.3)  184 (8.9)  1341 (64.6)  47 (2.3) | 521 (27.6)  208 (11.0)  1123 (59.4)  39 (2.1) | 91 (34.9)  24 (9.2)  136 (52.1)  10 (3.8) | 30 (53.6)  5 (8.9)  18 (32.1)  3 (5.4) | 6 (26.1)  3 (13.0)  12 (52.2)  2 (8.7) | <0.001 |
| Depth of sleep, *n* (%) |  |  |  |  |  |  |  |
| Deep  Fairly deep  Average  Fairly light  Light | 420 (22.4)  997 (53.2)  44 (2.3)  345 (18.4)  67 (3.6) | 402 (19.4)  1167 (56.2)  39 (1.9)  378 (18.2)  91 (4.4) | 263 (13.9)  1017 (53.8)  67 (3.5)  444 (23.5)  100 (5.3) | 26 (10.0)  123 (47.1)  9 (3.4)  82 (31.4)  21 (8.0) | 7 (12.5)  23 (41.1)  0 (0.0)  12 (21.4)  14 (25.0) | 2 (8.7)  12 (52.2)  1 (4.3)  5 (21.7)  3 (13.0) | <0.001 |
| Proximity of housing to the main road, *n* (%) |  |  |  |  |  |  |  |
| Along with road  ≤50 m  50 to ≤150 m  150 to ≤500 m  >500 m | 191 (10.2)  312 (16.7)  354 (18.9)  536 (28.6)  480 (25.6) | 268 (12.9)  384 (18.5)  424 (20.4)  558 (26.9)  443 (21.3) | 274 (14.5)  348 (18.4)  358 (18.9)  518 (27.4)  393 (20.8) | 34 (13.0)  40 (15.3)  46 (17.6)  75 (28.7)  66 (25.3) | 6 (10.7)  12 (21.4)  13 (23.2)  12 (21.4)  13 (23.2) | 3 (13.0)  1 (4.3)  3 (13.0)  5 (21.7)  11 (47.8) | 0.001 |
| Visibility of the main road, *n* (%) |  |  |  |  |  |  |  |
| Good  Partly  Poor | 190 (10.1)  382 (20.4)  1301 (69.5) | 235 (11.3)  538 (25.9)  1304 (62.8) | 244 (12.9)  508 (26.9)  1139 (60.2) | 36 (13.8)  63 (24.1)  162 (62.1) | 3 (5.4)  17 (30.4)  36 (64.3) | 3 (13.0)  4 (17.4)  16 (69.6) | <0.001 |
| Bedroom is close to main road (with), *n* (%) | 199 (10.6) | 305 (14.7) | 291 (15.4) | 37 (14.2) | 5 (8.9) | 2 (8.7) | <0.001 |
| Duration of residence, *n* (%) |  |  |  |  |  |  |  |
| <3 years  3 to <20 years  ≥ 20 years | 103 (5.5)  568 (30.3)  1202 (64.2) | 96 (4.6)  667 (32.1)  1314 (63.3) | 66 (3.5)  578 (30.6)  1247 (65.9) | 7 (2.7)  95 (36.4)  159 (60.9) | 2 (3.6)  14 (25.0)  40 (71.4) | 3 (13.0)  9 (39.1)  11 (47.8) | 0.015 |
| Housing structure, *n* (%) |  |  |  |  |  |  |  |
| Wooden  Reinforced concrete  Others | 1221 (65.2)  522 (27.9)  130 (6.9) | 1214 (58.4)  744 (35.8)  119 (5.7) | 1096 (58.0)  708 (37.4)  87 (4.6) | 165 (63.2)  88 (33.7)  8 (3.1) | 31 (55.4)  21 (37.5)  4 (7.1) | 13 (56.5)  9 (39.1)  1 (4.3) | <0.001 |
| Use of an unvented-heating appliance in winter (with), *n* (%) | 859 (45.9) | 967 (46.6) | 878 (46.4) | 112 (42.9) | 25 (44.6) | 12 (52.2) | 0.883 |
| Use of incense in the house (with), *n* (%) | 967 (51.6) | 1103 (53.1) | 1057 (55.9) | 137 (52.5) | 28 (50.0) | 14 (60.9) | 0.155 |

^a^ Data were missing for four participants. ^b^ The six levels were (1) no noise, (2) not annoyed, (3) less annoyed, (4) moderately annoyed, (5) annoyed, and (6) highly annoyed. Abbreviations: IHD, ischemic heart diseases. ^c^ *P* value from χ^2^ test.

**Table S1-e.** Baseline characteristics by noise exposure status (*n* = 6181).

| Potential confounder | Nocturnal neighborhood noise annoyance ^b^ | | | | | | *P* value ^c^ |
| --- | --- | --- | --- | --- | --- | --- | --- |
|  | (1) | (2) | (3) | (4) | (5) | (6) |  |
| Age, *n* (%) |  |  |  |  |  |  |  |
| <75 | 1595 (84.5) | 1800 (88.4) | 1557 (87.5) | 319 (90.4) | 80 (96.4) | 36 (87.8) | <0.001 |
| ≥75 | 292 (15.5) | 237 (11.6) | 223 (12.5) | 34 (9.6) | 3 (3.6) | 5 (12.2) |  |
| Male, *n* (%) | 984 (52.1) | 1032 (50.7) | 789 (44.3) | 154 (43.6) | 38 (45.8) | 21 (51.2) | <0.001 |
| Employment, *n* (%) |  |  |  |  |  |  |  |
| Employed | 142 (7.5) | 158 (7.8) | 124 (7.0) | 32 (9.1) | 7 (8.4) | 3 (7.3) | 0.134 |
| Self-employed | 168 (8.9) | 166 (8.1) | 164 (9.2) | 26 (7.4) | 6 (7.2) | 5 (12.2) |  |
| Housewife/househusband | 588 (31.2) | 671 (32.9) | 624 (35.1) | 120 (34.0) | 29 (34.9) | 11 (26.8) |  |
| Part-time employed | 126 (6.7) | 166 (8.1) | 153 (8.6) | 36 (10.2) | 9 (10.8) | 6 (14.6) |  |
| Unemployed | 645 (34.2) | 683 (33.5) | 546 (30.7) | 108 (30.6) | 27 (32.5) | 14 (34.1) |  |
| Others | 218 (11.6) | 193 (9.5) | 169 (9.5) | 31 (8.8) | 5 (6.0) | 2 (4.9) |  |
| Degree of obesity, *n* (%) ^a^ |  |  |  |  |  |  |  |
| Underweight | 128 (6.8) | 139 (6.8) | 132 (7.4) | 29 (8.2) | 6 (7.2) | 0 (0.0) | 0.449 |
| Normal | 1387 (73.5) | 1482 (72.8) | 1314 (73.9) | 277 (78.7) | 62 (74.7) | 32 (78.0) |  |
| Obesity (class I) | 337 (17.9) | 382 (18.8) | 296 (16.6) | 44 (12.5) | 15 (18.1) | 8 (19.5) |  |
| Obesity (class II) | 33 (1.7) | 28 (1.4) | 32 (1.8) | 2 (0.0) | 0 (0.0) | 1 (2.4) |  |
| Obesity (class III) | 2 (0.1) | 4 (0.2) | 3 (0.2) | 0 (0.0) | 0 (0.0) | 0 (0.0) |  |
| Obesity (class IV) | 0 (0.0) | 0 (0.0) | 2 (0.1) | 0 (0.0) | 0 (0.0) | 0 (0.0) |  |
| Medical history of parents (with), *n* (%) |  |  |  |  |  |  |  |
| Stroke  IHDs | 390 (20.7)  214 (11.3) | 453 (22.2)  230 (11.3) | 381 (21.4)  216 (12.1) | 72 (20.4)  47 (13.3) | 22 (26.5)  8 (9.6) | 4 (9.8)  9 (22.0) | 0.282  0.279 |
| Smoking status, *n* (%) |  |  |  |  |  |  |  |
| Current/every day | 198 (10.5) | 219 (10.8) | 196 (11.0) | 28 (7.9) | 6 (7.2) | 5 (12.2) | 0.011 |
| Current/sometimes | 20 (1.1) | 15 (0.7) | 24 (1.3) | 2 (0.6) | 0 (0.0) | 0 (0.0) |  |
| Former | 640 (33.9) | 670 (32.9) | 495 (27.8) | 109 (30.9) | 22 (26.5) | 15 (36.6) |  |
| Never | 1029 (54.5) | 1133 (55.6) | 1065 (59.8) | 214 (60.6) | 55 (66.3) | 21 (51.2) |  |
| Drinking habits, *n* (%) |  |  |  |  |  |  |  |
| Once or more a week | 886 (47.0) | 1012 (49.7) | 840 (47.2) | 166 (47.0) | 39 (47.0) | 19 (46.3) | 0.579 |
| Less than once a week or never | 1001 (53.0) | 1025 (50.3) | 940 (52.8) | 187 (53.0) | 44 (53.0) | 22 (53.7) |  |
| Regular exercise, *n* (%) |  |  |  |  |  |  |  |
| Current/always  Current/sometimes  Former  Never | 695 (36.8)  754 (40.0)  241 (12.8)  197 (10.4) | 743 (36.5)  862 (42.3)  243 (11.9)  189 (9.3) | 623 (35.0)  764 (42.9)  215 (12.1)  178 (10.0) | 111 (31.4)  165 (46.7)  42 (11.9)  35 (9.9) | 38 (45.8)  27 (32.5)  10 (12.0)  8 (9.6) | 19 (46.3)  15 (36.6)  4 (9.8)  3 (7.3) | 0.439 |
| Sufficiency of sleep duration, *n* (%) |  |  |  |  |  |  |  |
| Short  Average  Good  Long | 466 (24.7)  180 (9.5)  1194 (63.3)  47 (2.5) | 503 (24.7)  180 (8.8)  1310 (64.3)  44 (2.2) | 474 (26.6)  193 (10.8)  1074 (60.3)  39 (2.2) | 119 (33.7)  33 (9.3)  188 (53.3)  13 (3.7) | 34 (41.0)  8 (9.6)  38 (45.8)  3 (3.6) | 14 (34.1)  6 (14.6)  19 (46.3)  2 (4.9) | <0.001 |
| Depth of sleep, *n* (%) |  |  |  |  |  |  |  |
| Deep  Fairly deep  Average  Fairly light  Light | 420 (22.3)  1004 (53.2)  45 (2.4)  347 (18.4)  71 (3.8) | 404 (19.8)  1139 (55.9)  37 (1.8)  374 (18.4)  83 (4.1) | 245 (13.8)  983 (55.2)  67 (3.8)  393 (22.1)  92 (5.2) | 41 (11.6)  163 (46.2)  9 (2.5)  108 (30.6)  32 (9.1) | 7 (8.4)  32 (38.6)  1 (1.2)  33 (39.8)  10 (12.0) | 3 (7.3)  18 (43.9)  1 (2.4)  11 (26.8)  8 (19.5) | <0.001 |
| Proximity of housing to the main road, *n* (%) |  |  |  |  |  |  |  |
| Along with road  ≤50 m  50 to ≤150 m  150 to ≤500 m  >500 m | 188 (10.0)  312 (16.5)  358 (19.0)  548 (29.0)  481 (25.5) | 270 (13.3)  376 (18.5)  414 (20.3)  542 (26.6)  435 (21.4) | 252 (14.2)  320 (18.0)  338 (19.0)  498 (28.0)  372 (20.9) | 49 (13.9)  67 (19.0)  66 (18.7)  89 (25.2)  82 (23.2) | 10 (12.0)  20 (24.1)  17 (20.5)  12 (14.5)  24 (28.9) | 7 (17.1)  2 (4.9)  5 (12.2)  15 (36.6)  12 (29.3) | <0.001 |
| Visibility of the main road, *n* (%) |  |  |  |  |  |  |  |
| Good  Partly  Poor | 192 (10.2)  388 (20.6)  1307 (69.3) | 239 (11.7)  523 (25.7)  1275 (62.6) | 226 (12.7)  457 (25.7)  1097 (61.6) | 42 (11.9)  108 (30.6)  203 (57.5) | 7 (8.4)  28 (33.7)  48 (57.8) | 5 (12.2)  8 (19.5)  28 (68.3) | <0.001 |
| Bedroom is close to main road (with), *n* (%) | 205 (10.9) | 307 (15.1) | 265 (14.9) | 47 (13.3) | 12 (14.5) | 3 (7.3) | 0.001 |
| Duration of residence, *n* (%) |  |  |  |  |  |  |  |
| <3 years  3 to <20 years  ≥ 20 years | 100 (5.3)  586 (31.1)  1201 (63.6) | 95 (4.7)  646 (31.7)  1296 (63.6) | 65 (3.7)  543 (30.5)  1172 (65.8) | 8 (2.3)  122 (34.6)  223 (63.2) | 4 (4.8)  24 (28.9)  55 (66.3) | 5 (12.2)  10 (24.4)  26 (63.4) | 0.046 |
| Housing structure, *n* (%) |  |  |  |  |  |  |  |
| Wooden  Reinforced concrete  Others | 1234 (65.4)  523 (27.7)  130 (6.9) | 1213 (59.5)  708 (34.8)  116 (5.7) | 1033 (58.0)  662 (37.2)  85 (4.8) | 187 (53.0)  152 (43.1)  14 (4.0) | 50 (60.2)  29 (34.9)  4 (4.8) | 23 (56.1)  18 (43.9)  0 (0.0) | <0.001 |
| Use of an unvented-heating appliance in winter (with), *n* (%) | 867 (45.9) | 953 (46.8) | 830 (46.6) | 147 (41.6) | 36 (43.4) | 20 (48.8) | 0.581 |
| Use of incense in the house (with), *n* (%) | 971 (51.5) | 1097 (53.9) | 989 (55.6) | 180 (51.0) | 50 (60.2) | 19 (46.3) | 0.089 |

^a^ Data were missing for four participants. ^b^ The six levels were (1) no noise, (2) not annoyed, (3) less annoyed, (4) moderately annoyed, (5) annoyed, and (6) highly annoyed. Abbreviations: IHD, ischemic heart diseases. ^c^ *P* value from χ^2^ test.

**Table S1-f.** Baseline characteristics by noise exposure status (*n* = 6181).

| Potential confounder | Daytime construction noise annoyance ^b^ | | | | | | *P* value ^c^ |
| --- | --- | --- | --- | --- | --- | --- | --- |
|  | (1) | (2) | (3) | (4) | (5) | (6) |  |
| Age, *n* (%) |  |  |  |  |  |  |  |
| <75 | 1541 (870) | 985 (86.6) | 1845 (86.5) | 801 (88.5) | 150 (92.0) | 65 (90.3) | 0.245 |
| ≥75 | 230 (13.0) | 153 (13.4) | 287 (13.5) | 104 (11.5) | 13 (8.0) | 7 (9.7) |  |
| Male, *n* (%) | 914 (51.6) | 567 (49.8) | 992 (46.5) | 439 (48.5) | 73 (44.8) | 33 (45.8) | 0.038 |
| Employment, *n* (%) |  |  |  |  |  |  |  |
| Employed | 141 (8.0) | 100 (8.8) | 139 (6.5) | 72 (8.0) | 9 (5.5) | 5 (6.9) | 0.725 |
| Self-employed | 146 (8.2) | 88 (7.7) | 198 (9.3) | 83 (9.2) | 16 (9.8) | 4 (5.6) |  |
| Housewife/househusband | 558 (31.5) | 370 (32.5) | 742 (34.8) | 297 (32.8) | 51 (31.3) | 25 (34.7) |  |
| Part-time employed | 137 (7.7) | 95 (8.3) | 166 (7.8) | 77 (8.5) | 13 (8.0) | 8 (11.1) |  |
| Unemployed | 593 (33.5) | 375 (33.0) | 679 (31.8) | 296 (32.7) | 57 (35.0) | 23 (31.9) |  |
| Others | 196 (11.1) | 110 (9.7) | 208 (9.8) | 80 (8.8) | 17 (10.4) | 7 (9.7) |  |
| Degree of obesity, *n* (%) ^a^ |  |  |  |  |  |  |  |
| Underweight | 112 (6.3) | 77 (6.8) | 169 (7.9) | 65 (7.2) | 6 (3.7) | 5 (6.9) | 0.844 |
| Normal | 1298 (73.3) | 847 (74.5) | 1570 (73.7) | 660 (73.0) | 126 (77.3) | 53 (73.6) |  |
| Obesity (class I) | 334 (18.9) | 189 (16.6) | 360 (16.9) | 159 (17.6) | 28 (17.2) | 12 (16.7) |  |
| Obesity (class II) | 24 (1.4) | 21 (1.8) | 29 (1.4) | 17 (1.9) | 3 (1.8) | 2 (2.8) |  |
| Obesity (class III) | 2 (0.1) | 3 (0.3) | 2 (0.1) | 2 (0.2) | 0 (0.0) | 0 (0.0) |  |
| Obesity (class IV) | 0 (0.0) | 0 (0.0) | 1 (0.0) | 1 (0.1) | 0 (0.0) | 0 (0.0) |  |
| Medical history of parents (with), *n* (%) |  |  |  |  |  |  |  |
| Stroke  IHDs | 369 (20.8)  202 (11.4) | 260 (22.8)  128 (11.2) | 456 (21.4)  237 (11.1) | 190 (21.0)  127 (14.0) | 38 (23.3)  20 (12.3) | 9 (12.5)  10 (13.9) | 0.348  0.285 |
| Smoking status, *n* (%) |  |  |  |  |  |  |  |
| Current/every day | 177 (10.0) | 112 (9.8) | 235 (11.0) | 104 (11.5) | 16 (9.8) | 8 (11.1) | 0.488 |
| Current/sometimes | 21 (1.2) | 9 (0.8) | 20 (0.9) | 10 (1.1) | 1 (0.6) | 0 (0.0) |  |
| Former | 608 (34.3) | 343 (30.1) | 650 (30.5) | 281 (31.0) | 49 (30.1) | 20 (27.8) |  |
| Never | 965 (54.5) | 674 (59.2) | 1227 (57.6) | 510 (56.4) | 97 (59.5) | 44 (61.1) |  |
| Drinking habits, *n* (%) |  |  |  |  |  |  |  |
| Once or more a week | 819 (46.2) | 552 (48.5) | 1024 (48.0) | 461 (50.9) | 70 (42.9) | 36 (50.0) | 0.206 |
| Less than once a week or never | 952 (53.8) | 586 (51.5) | 1108 (52.0) | 444 (49.1) | 93 (57.1) | 36 (50.0) |  |
| Regular exercise, *n* (%) |  |  |  |  |  |  |  |
| Current/always  Current/sometimes  Former  Never | 661 (37.3)  701 (39.6)  221 (12.5)  188 (10.6) | 423 (37.2)  456 (40.1)  137 (12.0)  122 (10.7) | 741 (34.8)  933 (43.8)  259 (12.1)  199 (9.3) | 316 (34.9)  401 (44.3)  113 (12.5)  75 (8.3) | 62 (38.0)  73 (44.8)  14 (8.6)  14 (8.6) | 26 (36.1)  23 (31.9)  11 (15.3)  12 (16.7) | 0.113 |
| Sufficiency of sleep duration, *n* (%) |  |  |  |  |  |  |  |
| Short  Average  Good  Long | 455 (25.7)  160 (9.0)  1121 (63.3)  35 (2.0) | 263 (23.1)  98 (8.6)  750 (65.9)  27 (2.4) | 517 (24.2)  221 (10.4)  1345 (63.1)  49 (2.3) | 287 (31.7)  94 (10.4)  501 (55.4)  23 (2.5) | 63 (38.7)  17 (10.4)  73 (44.8)  10 (6.1) | 25 (34.7)  10 (13.9)  33 (45.8)  4 (5.6) | <0.001 |
| Depth of sleep, *n* (%) |  |  |  |  |  |  |  |
| Deep  Fairly deep  Average  Fairly light  Light | 394 (22.2)  926 (52.3)  44 (2.5)  335 (18.9)  72 (4.1) | 237 (20.8)  618 (54.3)  18 (1.6)  213 (18.7)  52 (4.6) | 337 (15.8)  1215 (57.0)  70 (3.3)  419 (19.7)  91 (4.3) | 126 (13.9)  470 (51.9)  21 (2.3)  235 (26.0)  53 (5.9) | 13 (8.0)  79 (48.5)  5 (3.1)  46 (28.2)  20 (12.3) | 13 (18.1)  31 (43.1)  2 (2.8)  18 (25.0)  8 (11.1) | <0.001 |
| Proximity of housing to the main road, *n* (%) |  |  |  |  |  |  |  |
| Along with road  ≤50 m  50 to ≤150 m  150 to ≤500 m  >500 m | 174 (9.8)  290 (16.4)  342 (19.3)  513 (29.0)  452 (25.5) | 144 (12.7)  180 (15.8)  237 (20.8)  315 (27.7)  262 (23.0) | 293 (13.7)  419 (19.7)  395 (18.5)  582 (27.3)  443 (20.8) | 122 (13.5)  171 (18.9)  180 (19.9)  246 (27.2)  186 (20.6) | 29 (17.8)  26 (16.0)  30 (18.4)  37 (22.7)  41 (25.2) | 14 (19.4)  11 (15.3)  14 (19.4)  11 (15.3)  22 (30.6) | <0.001 |
| Visibility of the main road, *n* (%) |  |  |  |  |  |  |  |
| Good  Partly  Poor | 176 (9.9)  365 (20.6)  1230 (69.5) | 110 (9.7)  288 (25.3)  740 (65.0) | 277 (13.0)  542 (25.4)  1313 (61.6) | 117 (12.9)  260 (28.7)  528 (58.3) | 23 (14.1)  36 (22.1)  104 (63.8) | 8 (11.1)  21 (29.2)  43 (59.7) | <0.001 |
| Bedroom is close to main road (with), *n* (%) | 189 (10.7) | 156 (13.7) | 309 (14.5) | 142 (15.7) | 34 (20.9) | 9 (12.5) | <0.001 |
| Duration of residence, *n* (%) |  |  |  |  |  |  |  |
| <3 years  3 to <20 years  ≥ 20 years | 98 (5.5)  564 (31.8)  1109 (62.6) | 63 (5.5)  376 (33.0)  699 (61.4) | 84 (3.9)  638 (29.9)  1410 (66.1) | 22 (2.4)  275 (30.4)  608 (67.2) | 5 (3.1)  49 (30.1)  109 (66.9) | 5 (6.9)  29 (40.3)  38 (52.8) | <0.001 |
| Housing structure, *n* (%) |  |  |  |  |  |  |  |
| Wooden  Reinforced concrete  Others | 1134 (64.0)  532 (30.0)  105 (5.9) | 683 (60.0)  391 (34.4)  64 (5.6) | 1265 (59.3)  750 (35.2)  117 (5.5) | 521 (57.6)  329 (36.4)  55 (6.1) | 99 (60.7)  59 (36.2)  5 (3.1) | 38 (52.8)  31 (43.1)  3 (4.2) | 0.018 |
| Use of an unvented-heating appliance in winter (with), *n* (%) | 830 (46.9) | 525 (46.1) | 997 (46.8) | 382 (42.2) | 76 (46.6) | 43 (59.7) | 0.039 |
| Use of incense in the house (with), *n* (%) | 898 (50.7) | 569 (50.0) | 1198 (56.2) | 517 (57.1) | 86 (52.8) | 38 (52.8) | <0.001 |

^a^ Data were missing for four participants. ^b^ The six levels were (1) no noise, (2) not annoyed, (3) less annoyed, (4) moderately annoyed, (5) annoyed, and (6) highly annoyed. Abbreviations: IHD, ischemic heart diseases. ^c^ *P* value from χ^2^ test.

**Table S1-g.** Baseline characteristics by noise exposure status (*n* = 6181).

| Potential confounder | Nocturnal construction noise annoyance ^b^ | | | | | | *P* value ^c^ |
| --- | --- | --- | --- | --- | --- | --- | --- |
|  | (1) | (2) | (3) | (4) | (5) | (6) |  |
| Age, *n* (%) |  |  |  |  |  |  |  |
| <75 | 1902 (87.2) | 1061 (87.3) | 1577 (86.1) | 562 (87.1) | 198 (92.5) | 87 (91.6) | 0.105 |
| ≥75 | 278 (12.8) | 154 (12.7) | 255 (83) | 83 (12.9) | 16 (7.5) | 8 (8.4) |  |
| Male, *n* (%) | 1077 (49.4) | 588 (48.4) | 880 (48.0) | 325 (50.4) | 103 (48.1) | 45 (47.4) | 0.902 |
| Employment, *n* (%) |  |  |  |  |  |  |  |
| Employed | 159 (7.3) | 101 (8.3) | 124 (6.8) | 58 (9.0) | 16 (7.5) | 8 (8.4) | 0.095 |
| Self-employed | 168 (7.7) | 94 (7.7) | 182 (9.9) | 63 (9.8) | 25 (11.7) | 3 (3.2) |  |
| Housewife/househusband | 714 (32.8) | 412 (33.9) | 621 (33.9) | 204 (31.6) | 61 (28.5) | 31 (32.6) |  |
| Part-time employed | 162 (7.4) | 99 (8.1) | 141 (7.7) | 61 (9.5) | 20 (9.3) | 13 (13.7) |  |
| Unemployed | 740 (33.9) | 390 (32.1) | 586 (32.0) | 198 (30.7) | 75 (35.0) | 34 (35.8) |  |
| Others | 237 (10.9) | 119 (9.8) | 178 (9.7) | 61 (9.5) | 17 (7.9) | 6 (6.3) |  |
| Degree of obesity, *n* (%) ^a^ |  |  |  |  |  |  |  |
| Underweight | 160 (7.3) | 83 (6.8) | 137 (7.5) | 30 (4.7) | 17 (7.9) | 7 (7.4) | 0.622 |
| Normal | 1598 (73.3) | 910 (75.0) | 1336 (73.0) | 486 (75.5) | 161 (75.2) | 63 (66.3) |  |
| Obesity (class I) | 389 (17.9) | 199 (16.4) | 324 (17.7) | 116 (18.0) | 32 (15.0) | 22 (23.2) |  |
| Obesity (class II) | 30 (1.4) | 18 (1.5) | 31 (1.7) | 10 (1.6) | 4 (1.9) | 3 (0.0) |  |
| Obesity (class III) | 2 (0.1) | 4 (0.3) | 2 (0.1) | 1 (0.2) | 0 (0.0) | 0 (0.0) |  |
| Obesity (class IV) | 0 (0.0) | 0 (0.0) | 1 (0.1) | 1 (0.2) | 0 (0.0) | 0 (0.0) |  |
| Medical history of parents (with), *n* (%) |  |  |  |  |  |  |  |
| Stroke  IHDs | 475 (21.8)  249 (11.4) | 277 (22.8)  145 (11.9) | 364 (19.9)  209 (11.4) | 151 (23.4)  83 (12.9) | 40 (18.7)  26 (12.1) | 15 (15.8)  12 (12.6) | 0.134  0.930 |
| Smoking status, *n* (%) |  |  |  |  |  |  |  |
| Current/every day | 215 (9.9) | 117 (9.6) | 215 (11.7) | 69 (10.7) | 24 (11.2) | 12 (12.6) | 0.367 |
| Current/sometimes | 21 (1.0) | 10 (0.8) | 19 (1.0) | 7 (1.1) | 3 (1.4) | 1 (1.1) |  |
| Former | 730 (33.5) | 359 (29.5) | 577 (31.5) | 195 (30.2) | 68 (31.8) | 22 (23.2) |  |
| Never | 1214 (55.7) | 729 (60.0) | 1021 (55.7) | 374 (58.0) | 119 (55.6) | 60 (63.2) |  |
| Drinking habits, *n* (%) |  |  |  |  |  |  |  |
| Once or more a week | 1018 (46.7) | 596 (49.1) | 873 (47.7) | 332 (51.5) | 100 (46.7) | 43 (45.3) | 0.343 |
| Less than once a week or never | 1162 (53.3) | 619 (50.9) | 959 (52.3) | 313 (48.5) | 114 (53.3) | 52 (54.7) |  |
| Regular exercise, *n* (%) |  |  |  |  |  |  |  |
| Current/always  Current/sometimes  Former  Never | 811 (37.2)  886 (40.6)  267 (12.2)  216 (9.9) | 452 (37.2)  486 (40.0)  154 (12.7)  123 (10.1) | 635 (34.7)  801 (43.7)  214 (11.7)  182 (9.9) | 221 (34.3)  282 (43.7)  81 (12.6)  61 (9.5) | 74 (34.6)  101 (47.2)  25 (11.7)  14 (6.5) | 36 (37.9)  31 (32.6)  14 (14.7)  14 (14.7) | 0.358 |
| Sufficiency of sleep duration, *n* (%) |  |  |  |  |  |  |  |
| Short  Average  Good  Long | 563 (25.8)  201 (9.2)  1374 (63.0)  42 (1.9) | 299 (24.6)  100 (8.2)  791 (65.1)  25 (2.1) | 442 (24.1)  191 (10.4)  1151 (62.8)  48 (2.6) | 192 (29.8)  70 (10.9)  365 (56.6)  18 (2.8) | 78 (36.4)  23 (10.7)  105 (49.1)  8 (3.7) | 36 (37.9)  15 (15.8)  37 (38.9)  7 (7.4) | <0.001 |
| Depth of sleep, *n* (%) |  |  |  |  |  |  |  |
| Deep  Fairly deep  Average  Fairly light  Light | 478 (21.9)  1153 (52.9)  49 (2.2)  413 (18.9)  87 (4.0) | 250 (20.6)  660 (54.3)  19 (1.6)  227 (18.7)  59 (4.9) | 272 (14.8)  1049 (57.3)  68 (3.7)  365 (19.9)  78 (4.3) | 92 (14.3)  326 (50.5)  11 (1.7)  172 (26.7)  44 (6.8) | 19 (8.9)  107 (50.0)  9 (4.2)  61 (28.5)  18 (8.4) | 9 (9.5)  44 (46.3)  4 (4.2)  28 (29.5)  10 (10.5) | <0.001 |
| Proximity of housing to the main road, *n* (%) |  |  |  |  |  |  |  |
| Along with road  ≤50 m  50 to ≤150 m  150 to ≤500 m  >500 m | 216 (9.9)  367 (16.8)  411 (18.9)  643 (29.5)  543 (24.9) | 140 (11.5)  197 (16.2)  249 (20.5)  339 (27.9)  290 (23.9) | 259 (14.1)  341 (18.6)  340 (18.6)  511 (27.9)  381 (20.8) | 98 (15.2)  133 (20.6)  139 (21.6)  152 (23.6)  123 (19.1) | 42 (19.6)  43 (20.1)  40 (18.7)  42 (19.6)  47 (22.0) | 21 (22.1)  16 (16.8)  19 (20.0)  17 (17.9)  22 (23.2) | <0.001 |
| Visibility of the main road, *n* (%) |  |  |  |  |  |  |  |
| Good  Partly  Poor | 204 (9.4)  466 (21.4)  1510 (69.3) | 110 (9.1)  286 (23.5)  819 (67.4) | 244 (13.3)  476 (26.0)  1112 (6.07) | 103 (16.0)  194 (30.1)  348 (54.0) | 35 (16.4)  62 (29.0)  117 (54.7) | 15 (15.8)  28 (29.5)  52 (54.7) | <0.001 |
| Bedroom is close to main road (with), *n* (%) | 240 (11.0) | 157 (12.9) | 275 (15.0) | 112 (17.4) | 41 (19.2) | 14 (14.7) | <0.001 |
| Duration of residence, *n* (%) |  |  |  |  |  |  |  |
| <3 years  3 to <20 years  ≥ 20 years | 114 (5.2)  691 (31.7)  1375 (63.1) | 61 (5.0)  409 (33.7)  745 (61.3) | 75 (4.1)  538 (29.4)  1219 (66.5) | 13 (2.0)  196 (30.4)  436 (67.6) | 9 (4.2)  61 (28.5)  144 (67.3) | 5 (5.3)  36 (37.9)  54 (56.8) | 0.005 |
| Housing structure, *n* (%) |  |  |  |  |  |  |  |
| Wooden  Reinforced concrete  Others | 1371 (62.9)  671 (30.8)  138 (6.3) | 730 (60.1)  419 (34.5)  66 (5.4) | 1085 (59.2)  647 (35.3)  100 (5.5) | 371 (57.5)  243 (37.7)  31 (4.8) | 129 (60.3)  74 (34.6)  11 (5.1) | 54 (56.8)  38 (40.0)  3 (3.2) | 0.041 |
| Use of an unvented-heating appliance in winter (with), *n* (%) | 1017 (46.7) | 544 (44.8) | 863 (47.1) | 283 (43.9) | 95 (44.4) | 51 (53.7) | 0.347 |
| Use of incense in the house (with), *n* (%) | 1120 (51.4) | 612 (50.4) | 1028 (56.1) | 374 (58.0) | 117 (54.7) | 55 (57.9) | 0.001 |

^a^ Data were missing for four participants. ^b^ The six levels were (1) no noise, (2) not annoyed, (3) less annoyed, (4) moderately annoyed, (5) annoyed, and (6) highly annoyed. Abbreviations: IHD, ischemic heart diseases. ^c^ *P* value from χ^2^ test.

**Table S1-h.** Baseline characteristics by noise exposure status (*n* = 6181).

| Potential confounder | Daytime railway noise annoyance ^b^ | | | | | | *P* value ^c^ |
| --- | --- | --- | --- | --- | --- | --- | --- |
|  | (1) | (2) | (3) | (4) | (5) | (6) |  |
| Age, *n* (%) |  |  |  |  |  |  |  |
| <75 | 3024 (87.0) | 1256 (86.4) | 988 (88.3) | 86 (87.8) | 20 (90.9) | 13 (92.9) | 0.742 |
| ≥75 | 451 (13.0) | 197 (13.6) | 131 (11.7) | 12 (12.2) | 2 (9.1) | 1 (7.1) |  |
| Male, *n* (%) | 1699 (48.9) | 736 (50.7) | 528 (47.2) | 44 (44.9) | 4 (18.2) | 7 (50.0) | 0.034 |
| Employment, *n* (%) |  |  |  |  |  |  |  |
| Employed | 256 (7.4) | 121 (8.3) | 79 (7.1) | 8 (8.2) | 1 (4.5) | 1 (7.1) | 0.092 |
| Self-employed | 303 (8.7) | 119 (8.2) | 95 (8.5) | 18 (18.4) | 0 (0.0) | 0 (0.0) |  |
| Housewife/househusband | 1177 (33.9) | 458 (31.5) | 361 (32.3) | 30 (30.6) | 14 (63.6) | 3 (21.4) |  |
| Part-time employed | 275 (7.9) | 116 (8.0) | 96 (8.6) | 6 (6.1) | 1 (4.5) | 2 (14.3) |  |
| Unemployed | 1127 (32.4) | 499 (34.3) | 358 (32.0) | 28 (28.6) | 5 (22.7) | 6 (42.9) |  |
| Others | 337 (9.7) | 140 (9.6) | 130 (11.6) | 8 (8.2) | 1 (4.5) | 2 (14.3) |  |
| Degree of obesity, *n* (%) ^a^ |  |  |  |  |  |  |  |
| Underweight | 244 (7.0) | 99 (6.8) | 83 (7.4) | 7 (7.1) | 1 (4.5) | 0 (0.0) | 0.333 |
| Normal | 2558 (73.6) | 1063 (73.2) | 836 (74.8) | 69 (70.4) | 15 (68.2) | 13 (92.9) |  |
| Obesity (class I) | 614 (17.7) | 257 (17.7) | 184 (16.5) | 22 (22.4) | 4 (18.2) | 1 (7.1) |  |
| Obesity (class II) | 54 (1.6) | 26 (1.8) | 14 (1.3) | 0 (0.0) | 2 (9.1) | 0 (0.0) |  |
| Obesity (class III) | 3 (0.1) | 6 (0.4) | 0 (0.0) | 0 (0.0) | 0 (0.0) | 0 (0.0) |  |
| Obesity (class IV) | 1 (0.0) | 1 (0.1) | 0 (0.0) | 0 (0.0) | 0 (0.0) | 0 (0.0) |  |
| Medical history of parents (with), *n* (%) |  |  |  |  |  |  |  |
| Stroke  IHDs | 767 (22.1)  419 (12.1) | 308 (21.2)  166 (11.4) | 215 (19.2)  124 (11.1) | 25 (25.5)  11 (11.2) | 6 (27.3)  1 (4.5) | 1 (7.1)  3 (21.4) | 0.201  0.648 |
| Smoking status, *n* (%) |  |  |  |  |  |  |  |
| Current/every day | 355 (10.2) | 158 (10.9) | 126 (11.3) | 11 (11.2) | 1 (4.5) | 1 (7.1) | 0.657 |
| Current/sometimes | 29 (0.8) | 15 (1.0) | 15 (1.3) | 1 (1.0) | 1 (4.5) | 0 (0.0) |  |
| Former | 1132 (32.6) | 454 (31.2) | 326 (29.1) | 29 (29.6) | 5 (22.7) | 5 (35.7) |  |
| Never | 1959 (56.4) | 826 (56.8) | 652 (58.3) | 57 (58.2) | 15 (68.2) | 8 (57.1) |  |
| Drinking habits, *n* (%) |  |  |  |  |  |  |  |
| Once or more a week | 1637 (47.1) | 735 (50.6) | 518 (46.3) | 55 (56.1) | 9 (40.9) | 8 (57.1) | 0.081 |
| Less than once a week or never | 1838 (52.9) | 718 (49.4) | 601 (53.7) | 43 (43.9) | 13 (59.1) | 6 (42.9) |  |
| Regular exercise, *n* (%) |  |  |  |  |  |  |  |
| Current/always  Current/sometimes  Former  Never | 1251 (36.0)  1451 (41.8)  436 (12.5)  337 (9.7) | 553 (38.1)  590 (40.6)  173 (11.9)  137 (9.4) | 385 (34.4)  483 (43.2)  131 (11.7)  120 (10.7) | 33 (33.7)  48 (49.0)  8 (8.2)  9 (9.2) | 4 (18.2)  7 (31.8)  7 (31.8)  4 (18.2) | 3 (21.4)  8 (57.1)  0 (0.0)  3 (21.4) | 0.061 |
| Sufficiency of sleep duration, *n* (%) |  |  |  |  |  |  |  |
| Short  Average  Good  Long | 910 (26.2)  322 (9.3)  2167 (62.4)  76 (2.2) | 350 (24.1)  133 (9.2)  929 (63.9)  41 (2.8) | 301 (26.9)  131 (11.7)  662 (59.2)  25 (2.2) | 34 (34.7)  11 (11.2)  50 (51.0)  3 (3.1) | 8 (36.4)  2 (9.1)  11 (50.0)  1 (4.5) | 7 (50.0)  1 (7.1)  4 (28.6)  2 (14.3) | 0.004 |
| Depth of sleep, *n* (%) |  |  |  |  |  |  |  |
| Deep  Fairly deep  Average  Fairly light  Light | 674 (19.4)  1860 (53.5)  85 (2.4)  697 (20.1)  159 (4.6) | 262 (18.0)  824 (56.7)  30 (2.1)  270 (18.6)  67 (4.6) | 165 (14.7)  590 (52.7)  40 (3.6)  266 (23.8)  58 (5.2) | 12 (12.2)  52 (53.1)  2 (2.0)  25 (25.5)  7 (7.1) | 5 (22.7)  7 (31.8)  2 (9.1)  6 (27.3)  2 (9.1) | 2 (14.3)  6 (42.9)  1 (7.1)  2 (14.3)  3 (21.4) | <0.001 |
| Proximity of housing to the main road, *n* (%) |  |  |  |  |  |  |  |
| Along with road  ≤50 m  50 to ≤150 m  150 to ≤500 m  >500 m | 431 (12.4)  652 (18.8)  653 (18.8)  927 (26.7)  812 (23.4) | 179 (12.3)  236 (16.2)  313 (21.5)  408 (28.1)  317 (21.8) | 144 (12.9)  195 (17.4)  201 (18.0)  337 (30.1)  242 (21.6) | 16 (16.3)  10 (10.2)  24 (24.5)  26 (26.5)  22 (22.4) | 3 (13.6)  3 (13.6)  5 (22.7)  5 (22.7)  6 (27.3) | 3 (21.4)  1 (7.1)  2 (14.3)  1 (7.1)  7 (50.0) | 0.070 |
| Visibility of the main road, *n* (%) |  |  |  |  |  |  |  |
| Good  Partly  Poor | 377 (10.8)  837 (24.1)  2261 (65.1) | 164 (11.3)  370 (25.5)  919 (63.2) | 142 (12.7)  270 (24.1)  707 (63.2) | 20 (20.4)  26 (26.5)  52 (53.1) | 7 (31.8)  4 (18.2)  11 (50.0) | 1 (7.1)  5 (35.7)  8 (57.1) | 0.010 |
| Bedroom is close to main road (with), *n* (%) | 450 (12.9) | 207 (14.2) | 163 (14.6) | 14 (14.3) | 3 (13.6) | 2 (14.3) | 0.746 |
| Duration of residence, *n* (%) |  |  |  |  |  |  |  |
| <3 years  3 to <20 years  ≥ 20 years | 159 (4.6)  1081 (31.1)  2235 (64.3) | 64 (4.4)  463 (31.9)  926 (63.7) | 50 (4.5)  334 (29.8)  735 (65.7) | 4 (4.1)  35 (35.7)  59 (60.2) | 0 (0.0)  10 (45.5)  12 (54.5) | 0 (0.0)  8 (57.1)  6 (42.9) | 0.469 |
| Housing structure, *n* (%) |  |  |  |  |  |  |  |
| Wooden  Reinforced concrete  Others | 2171 (62.5)  1086 (31.3)  218 (6.3) | 855 (58.8)  518 (35.7)  80 (5.5) | 641 (57.3)  432 (38.6)  46 (4.1) | 56 (57.1)  37 (37.8)  5 (5.1) | 11 (50.0)  11 (50.0)  0 (0.0) | 6 (42.9)  8 (57.1)  0 (0.0) | <0.001 |
| Use of an unvented-heating appliance in winter (with), *n* (%) | 1654 (47.6) | 682 (46.9) | 465 (41.6) | 33 (33.7) | 12 (54.5) | 7 (50.0) | 0.001 |
| Use of incense in the house (with), *n* (%) | 1858 (53.5) | 766 (52.7) | 611 (54.6) | 53 (54.1) | 11 (50.0) | 7 (50.0) | 0.955 |

^a^ Data were missing for four participants. ^b^ The six levels were (1) no noise, (2) not annoyed, (3) less annoyed, (4) moderately annoyed, (5) annoyed, and (6) highly annoyed. Abbreviations: IHD, ischemic heart diseases. ^c^ *P* value from χ^2^ test.

**Table S1-i.** Baseline characteristics by noise exposure status (*n* = 6181).

| Potential confounder | Nocturnal railway noise annoyance ^b^ | | | | | | *P* value ^c^ |
| --- | --- | --- | --- | --- | --- | --- | --- |
|  | (1) | (2) | (3) | (4) | (5) | (6) |  |
| Age, *n* (%) |  |  |  |  |  |  |  |
| <75 | 3026 (87.0) | 1221 (86.7) | 979 (87.7) | 119 (88.1) | 24 (92.3) | 18 (94.7) | 0.803 |
| ≥75 | 451 (13.0) | 187 (13.3) | 137 (12.3) | 16 (11.9) | 2 (7.7) | 1 (5.3) |  |
| Male, *n* (%) | 1691 (48.6) | 720 (51.1) | 520 (596) | 73 (54.1) | 8 (30.8) | 6 (31.6) | 0.029 |
| Employment, *n* (%) |  |  |  |  |  |  |  |
| Employed | 257 (7.4) | 116 (8.2) | 79 (7.1) | 10 (7.4) | 2 (7.7) | 2 (10.5) | 0.442 |
| Self-employed | 307 (8.8) | 113 (8.0) | 93 (8.3) | 18 (13.3) | 3 (11.5) | 1 (5.3) |  |
| Housewife/househusband | 1178 (33.9) | 442 (31.4) | 367 (32.9) | 37 (27.4) | 13 (50.0) | 6 (31.6) |  |
| Part-time employed | 278 (8.0) | 114 (8.1) | 95 (8.5) | 5 (3.7) | 1 (3.8) | 3 (15.8) |  |
| Unemployed | 1121 (32.2) | 486 (34.5) | 356 (31.9) | 50 (37.0) | 4 (15.4) | 6 (31.6) |  |
| Others | 336 (9.7) | 137 (9.7) | 126 (11.3) | 15 (11.1) | 3 (11.5) | 1 (5.3) |  |
| Degree of obesity, *n* (%) ^a^ |  |  |  |  |  |  |  |
| Underweight | 247 (7.1) | 90 (6.4) | 85 (7.6) | 11 (8.1) | 1 (3.8) | 0 (0.0) | 0.726 |
| Normal | 2556 (73.5) | 1032 (73.3) | 829 (74.4) | 100 (74.1) | 20 (76.9) | 17 (89.5) |  |
| Obesity (class I) | 616 (17.7) | 251 (17.8) | 186 (16.7) | 23 (17.0) | 4 (15.4) | 2 (10.5) |  |
| Obesity (class II) | 53 (1.5) | 27 (1.9) | 14 (1.3) | 1 (0.7) | 1 (3.8) | 0 (0.0) |  |
| Obesity (class III) | 3 (0.1) | 6 (0.4) | 0 (0.0) | 0 (0.0) | 0 (0.0) | 0 (0.0) |  |
| Obesity (class IV) | 1 (0.0) | 1 (0.1) | 0 (0.0) | 0 (0.0) | 0 (0.0) | 0 (0.0) |  |
| Medical history of parents (with), *n* (%) |  |  |  |  |  |  |  |
| Stroke  IHDs | 772 (22.2)  424 (12.2) | 293 (20.8)  153 (10.9) | 211 (18.9)  126 (11.3) | 38 (28.1)  14 (10.4) | 5 (19.2)  2 (7.7) | 3 (15.8)  5 (26.3) | 0.080  0.260 |
| Smoking status, *n* (%) |  |  |  |  |  |  |  |
| Current/every day | 357 (10.3) | 152 (10.8) | 128 (11.5) | 12 (8.9) | 2 (7.7) | 1 (5.3) | 0.093 |
| Current/sometimes | 29 (0.8) | 15 (1.1) | 11 (1.0) | 5 (3.7) | 1 (3.8) | 0 (0.0) |  |
| Former | 1123 (32.3) | 449 (31.9) | 319 (28.6) | 48 (35.6) | 7 (26.9) | 5 (26.3) |  |
| Never | 1968 (56.6) | 792 (56.3) | 658 (59.0) | 70 (51.9) | 16 (61.5) | 13 (68.4) |  |
| Drinking habits, *n* (%) |  |  |  |  |  |  |  |
| Once or more a week | 1636 (47.1) | 710 (50.4) | 525 (47.0) | 69 (51.1) | 14 (53.8) | 8 (42.1) | 0.296 |
| Less than once a week or never | 1841 (52.9) | 698 (49.6) | 591 (53.0) | 66 (48.9) | 12 (46.2) | 11 (57.9) |  |
| Regular exercise, *n* (%) |  |  |  |  |  |  |  |
| Current/always  Current/sometimes  Former  Never | 1252 (36.0)  1449 (41.7)  443 (12.7)  333 (9.6) | 537 (38.1)  566 (40.2)  164 (11.6)  141 (10.0) | 385 (34.5)  486 (43.5)  130 (11.6)  115 (10.3) | 44 (32.6)  66 (48.9)  11 (8.1)  14 (10.4) | 6 (23.1)  9 (34.6)  7 (26.9)  4 (15.4) | 5 (26.3)  11 (57.9)  0 (0.0)  3 (15.8) | 0.117 |
| Sufficiency of sleep duration, *n* (%) |  |  |  |  |  |  |  |
| Short  Average  Good  Long | 911 (26.2)  324 (9.3)  2167 (62.3)  75 (2.2) | 343 (24.4)  127 (9.0)  897 (63.7)  41 (2.9) | 296 (26.5)  125 (11.2)  670 (60.0)  25 (2.2) | 45 (33.3)  20 (14.8)  67 (49.6)  3 (2.2) | 6 (23.1)  3 (11.5)  15 (57.7)  2 (7.7) | 9 (47.4)  1 (5.3)  7 (36.8)  2 (10.5) | 0.004 |
| Depth of sleep, *n* (%) |  |  |  |  |  |  |  |
| Deep  Fairly deep  Average  Fairly light  Light | 670 (19.3)  1863 (53.6)  83 (2.4)  702 (20.2)  159 (4.6) | 266 (18.9)  796 (56.5)  28 (2.0)  250 (17.8)  68 (4.8) | 162 (14.5)  597 (53.5)  41 (3.7)  259 (23.2)  57 (5.1) | 12 (8.9)  67 (49.6)  5 (3.7)  44 (32.6)  7 (5.2) | 6 (23.1)  10 (38.5)  2 (7.7)  5 (19.2)  3 (11.5) | 4 (21.1)  6 (31.6)  1 (5.3)  6 (31.6)  2 (10.5) | <0.001 |
| Proximity of housing to the main road, *n* (%) |  |  |  |  |  |  |  |
| Along with road  ≤50 m  50 to ≤150 m  150 to ≤500 m  >500 m | 433 (12.5)  653 (18.8)  654 (18.8)  926 (26.6)  811 (23.3) | 177 (12.6)  226 (16.1)  293 (20.8)  402 (28.6)  310 (22.0) | 141 (12.6)  190 (17.0)  207 (18.5)  331 (29.7)  247 (22.1) | 17 (12.6)  23 (17.0)  35 (25.9)  36 (26.7)  24 (17.8) | 5 (19.2)  4 (15.4)  6 (23.1)  6 (23.1)  5 (19.2) | 3 (15.8)  1 (5.3)  3 (15.8)  3 (15.8)  9 (47.4) | 0.179 |
| Visibility of the main road, *n* (%) |  |  |  |  |  |  |  |
| Good  Partly  Poor | 382 (11.0)  844 (24.3)  2251 (64.7) | 156 (11.1)  341 (24.2)  911 (64.7) | 133 (11.9)  277 (24.8)  706 (63.3) | 26 (19.3)  40 (29.6)  69 (51.1) | 10 (38.5)  3 (11.5)  13 (50.0) | 4 (21.1)  7 (36.8)  8 (42.1) | <0.001 |
| Bedroom is close to main road (with), *n* (%) | 452 (13.0) | 196 (13.9) | 164 (14.7) | 19 (14.1) | 4 (15.4) | 4 (21.1) | 0.650 |
| Duration of residence, *n* (%) |  |  |  |  |  |  |  |
| <3 years  3 to <20 years  ≥ 20 years | 160 (4.6)  1081 (31.1)  2236 (64.3) | 58 (4.1)  447 (31.7)  903 (64.1) | 54 (4.8)  332 (29.7)  730 (65.4) | 4 (3.0)  52 (38.5)  79 (58.5) | 1 (3.8)  9 (34.6)  16 (61.5) | 0 (0.0)  10 (52.6)  9 (47.4) | 0.413 |
| Housing structure, *n* (%) |  |  |  |  |  |  |  |
| Wooden  Reinforced concrete  Others | 2166 (62.3)  1093 (31.4)  218 (6.3) | 838 (59.5)  491 (34.9)  79 (5.6) | 633 (56.7)  436 (39.1)  47 (4.2) | 82 (60.7)  48 (35.6)  5 (3.7) | 13 (50.0)  13 (50.0)  0 (0.0) | 8 (42.1)  11 (57.9)  0 (0.0) | <0.001 |
| Use of an unvented-heating appliance in winter (with), *n* (%) | 1655 (47.6) | 657 (46.7) | 478 (42.8) | 41 (30.4) | 14 (53.8) | 8 (42.1) | <0.001 |
| Use of incense in the house (with), *n* (%) | 1863 (53.6) | 746 (53.0) | 599 (53.7) | 74 (54.8) | 15 (57.7) | 9 (47.4) | 0.981 |

^a^ Data were missing for four participants. ^b^ The six levels were (1) no noise, (2) not annoyed, (3) less annoyed, (4) moderately annoyed, (5) annoyed, and (6) highly annoyed. Abbreviations: IHD, ischemic heart diseases. ^c^ *P* value from χ^2^ test.

**Table S1-j.** Baseline characteristics by noise exposure status (*n* = 6181).

| Potential confounder | Daytime aircraft noise annoyance ^b^ | | | | | | *P* value ^c^ |
| --- | --- | --- | --- | --- | --- | --- | --- |
|  | (1) | (2) | (3) | (4) | (5) | (6) |  |
| Age, *n* (%) |  |  |  |  |  |  |  |
| <75 | 2017 (88.9) | 1443 (87.7) | 1377 (84.2) | 362 (87.2) | 127 (90.7) | 61 (80.3) | <0.001 |
| ≥75 | 253 (11.1) | 202 (12.3) | 258 (15.8) | 53 (12.8) | 13 (9.3) | 15 (19.7) |  |
| Male, *n* (%) | 984 (43.3) | 865 (52.6) | 831 (50.8) | 215 (51.8) | 75 (53.6) | 48 (63.2) | <0.001 |
| Employment, *n* (%) |  |  |  |  |  |  |  |
| Employed | 173 (7.6) | 137 (8.3) | 111 (6.8) | 30 (7.2) | 14 (10.0) | 1 (1.3) | <0.001 |
| Self-employed | 193 (8.5) | 170 (10.3) | 124 (7.6) | 35 (8.4) | 10 (7.1) | 3 (3.9) |  |
| Housewife/househusband | 807 (35.6) | 511 (31.1) | 542 (33.1) | 121 (29.2) | 42 (30.0) | 20 (26.3) |  |
| Part-time employed | 186 (8.2) | 121 (7.4) | 129 (7.9) | 38 (9.2) | 17 (12.1) | 5 (6.6) |  |
| Unemployed | 704 (31.0) | 557 (33.9) | 532 (32.5) | 146 (35.2) | 44 (31.4) | 40 (52.6) |  |
| Others | 207 (9.1) | 149 (9.1) | 197 (12.0) | 45 (10.8) | 13 (9.3) | 7 (9.2) |  |
| Degree of obesity, *n* (%) ^a^ |  |  |  |  |  |  |  |
| Underweight | 167 (7.4) | 101 (6.1) | 118 (7.2) | 29 (7.0) | 13 (9.3) | 6 (7.9) | 0.771 |
| Normal | 1680 (74.0) | 1206 (73.4) | 1206 (73.9) | 302 (72.8) | 101 (72.1) | 59 (77.6) |  |
| Obesity (class I) | 380 (16.7) | 309 (18.8) | 283 (17.3) | 80 (19.3) | 22 (15.7) | 8 (10.5) |  |
| Obesity (class II) | 37 (1.6) | 25 (1.5) | 24 (1.5) | 4 (1.0) | 3 (2.1) | 3 (3.9) |  |
| Obesity (class III) | 4 (0.2) | 3 (0.2) | 1 (0.1) | 0 (0.0) | 1 (0.7) | 0 (0.0) |  |
| Obesity (class IV) | 1 (0.0) | 0 (0.0) | 1 (0.1) | 0 (0.0) | 0 (0.0) | 0 (0.0) |  |
| Medical history of parents (with), *n* (%) |  |  |  |  |  |  |  |
| Stroke  IHDs | 481 (21.2)  264 (11.6) | 355 (21.6)  196 (11.9) | 347 (21.2)  190 (11.6) | 89 (21.4)  47 (11.3) | 38 (27.1)  21 (15.0) | 12 (15.8)  6 (7.9) | 0.508  0.748 |
| Smoking status, *n* (%) |  |  |  |  |  |  |  |
| Current/every day | 230 (10.1) | 178 (10.8) | 186 (11.4) | 36 (8.7) | 12 (8.6) | 10 (13.2) | 0.031 |
| Current/sometimes | 21 (0.9) | 14 (0.9) | 19 (1.2) | 5 (1.2) | 1 (0.7) | 1 (1.3) |  |
| Former | 666 (29.3) | 537 (32.6) | 512 (31.3) | 146 (35.2) | 56 (40.0) | 34 (44.7) |  |
| Never | 1353 (59.6) | 916 (55.7) | 918 (56.1) | 228 (54.9) | 71 (50.7) | 31 (40.8) |  |
| Drinking habits, *n* (%) |  |  |  |  |  |  |  |
| Once or more a week | 1026 (45.2) | 862 (52.4) | 763 (46.7) | 197 (47.5) | 72 (51.4) | 42 (55.3) | <0.001 |
| Less than once a week or never | 1244 (54.8) | 783 (47.6) | 872 (53.3) | 218 (52.5) | 68 (48.6) | 34 (44.7) |  |
| Regular exercise, *n* (%) |  |  |  |  |  |  |  |
| Current/always  Current/sometimes  Former  Never | 814 (35.9)  935 (41.2)  289 (12.7)  232 (10.2) | 625 (38.0)  671 (40.8)  202 (12.3)  147 (8.9) | 581 (35.5)  706 (43.2)  192 (11.7)  156 (9.5) | 130 (31.3)  186 (44.8)  50 (12.0)  49 (11.8) | 49 (35.0)  65 (46.4)  11 (7.9)  15 (10.7) | 30 (39.5)  24 (31.6)  11 (14.5)  11 (14.5) | 0.257 |
| Sufficiency of sleep duration, *n* (%) |  |  |  |  |  |  |  |
| Short  Average  Good  Long | 583 (25.7)  226 (10.0)  1422 (62.6)  39 (1.7) | 416 (25.3)  132 (8.0)  1053 (64.0)  44 (2.7) | 409 (25.0)  180 (11.0)  1006 (61.5)  40 (2.4) | 117 (28.2)  44 (10.6)  240 (57.8)  14 (3.4) | 49 (35.0)  14 (10.0)  71 (50.7)  6 (4.3) | 36 (47.4)  4 (5.3)  31 (40.8)  5 (6.6) | <0.001 |
| Depth of sleep, *n* (%) |  |  |  |  |  |  |  |
| Deep  Fairly deep  Average  Fairly light  Light | 455 (20.0)  1227 (54.1)  56 (2.5)  431 (19.0)  101 (4.4) | 296 (18.0)  942 (57.3)  26 (1.6)  310 (18.8)  71 (4.3) | 281 (17.2)  863 (52.8)  54 (3.3)  356 (21.8)  81 (5.0) | 67 (16.1)  207 (49.9)  16 (3.9)  104 (25.1)  21 (5.1) | 12 (8.6)  68 (48.6)  4 (2.9)  46 (32.9)  10 (7.1) | 9 (11.8)  32 (42.1)  4 (5.3)  19 (25.0)  12 (15.8) | <0.001 |
| Proximity of housing to the main road, *n* (%) |  |  |  |  |  |  |  |
| Along with road  ≤50 m  50 to ≤150 m  150 to ≤500 m  >500 m | 296 (13.0)  411 (18.1)  428 (18.9)  620 (27.3)  515 (22.7) | 227 (13.8)  291 (17.7)  344 (20.9)  418 (25.4)  365 (22.2) | 197 (12.0)  284 (17.4)  308 (18.8)  468 (28.6)  378 (23.1) | 37 (8.9)  69 (16.6)  80 (19.3)  131 (31.6)  98 (23.6) | 13 (9.3)  25 (17.9)  23 (16.4)  44 (31.4)  35 (25.0) | 6 (7.9)  17 (22.4)  15 (19.7)  23 (30.3)  15 (19.7) | 0.324 |
| Visibility of the main road, *n* (%) |  |  |  |  |  |  |  |
| Good  Partly  Poor | 270 (11.9)  561 (24.7)  1439 (63.4) | 198 (12.0)  394 (24.0)  1053 (64.0) | 184 (11.3)  410 (25.1)  1041 (63.7) | 44 (10.6)  86 (20.7)  285 (68.7) | 13 (9.3)  37 (26.4)  90 (64.3) | 2 (2.6)  24 (31.6)  50 (65.8) | 0.215 |
| Bedroom is close to main road (with), *n* (%) | 285 (12.6) | 233 (14.2) | 228 (13.9) | 63 (15.2) | 19 (13.6) | 11 (14.5) | 0.600 |
| Duration of residence, *n* (%) |  |  |  |  |  |  |  |
| <3 years  3 to <20 years  ≥ 20 years | 125 (5.5)  726 (32.0)  1419 (62.5) | 73 (4.4)  536 (32.6)  1036 (63.0) | 59 (3.6)  484 (29.6)  1092 (66.8) | 10 (2.4)  129 (31.1)  276 (66.5) | 5 (3.6)  34 (24.3)  101 (72.1) | 5 (6.6)  22 (28.9)  49 (64.5) | 0.011 |
| Housing structure, *n* (%) |  |  |  |  |  |  |  |
| Wooden  Reinforced concrete  Others | 1356 (59.7)  782 (34.4)  132 (5.8) | 986 (59.9)  564 (34.3)  95 (5.8) | 986 (60.3)  567 (34.7)  82 (5.0) | 275 (66.3)  115 (27.7)  25 (6.0) | 90 (64.3)  42 (30.0)  8 (5.7) | 47 (61.8)  22 (28.9)  7 (9.2) | 0.252 |
| Use of an unvented-heating appliance in winter (with), *n* (%) | 1039 (45.8) | 791 (48.1) | 723 (44.2) | 194 (46.7) | 77 (55.0) | 29 (38.2) | 0.043 |
| Use of incense in the house (with), *n* (%) | 1196 (52.7) | 894 (54.3) | 884 (54.1) | 224 (54.0) | 73 (52.1) | 35 (46.1) | 0.681 |

^a^ Data were missing for four participants. ^b^ The six levels were (1) no noise, (2) not annoyed, (3) less annoyed, (4) moderately annoyed, (5) annoyed, and (6) highly annoyed. Abbreviations: IHD, ischemic heart diseases. ^c^ *P* value from χ^2^ test.

**Table S1-k.** Baseline characteristics by noise exposure status (*n* = 6181).

| Potential confounder | Nocturnal aircraft noise annoyance ^b^ | | | | | | *P* value ^c^ |
| --- | --- | --- | --- | --- | --- | --- | --- |
|  | (1) | (2) | (3) | (4) | (5) | (6) |  |
| Age, *n* (%) |  |  |  |  |  |  |  |
| <75 | 2153 (88.9) | 1434 (87.2) | 1283 (84.7) | 319 (86.2) | 122 (87.1) | 76 (84.4) | 0.009 |
| ≥75 | 269 (11.1) | 211 (12.8) | 231 (15.3) | 51 (13.8) | 18 (12.9) | 14 (15.6) |  |
| Male, *n* (%) | 1045 (43.1) | 858 (52.2) | 787 (52.0) | 200 (54.1) | 75 (53.6) | 53 (58.9) | <0.001 |
| Employment, *n* (%) |  |  |  |  |  |  |  |
| Employed | 180 (7.4) | 138 (8.4) | 101 (6.7) | 27 (7.3) | 15 (10.7) | 5 (5.6) | <0.001 |
| Self-employed | 202 (8.3) | 169 (10.3) | 117 (7.7) | 33 (8.9) | 10 (7.1) | 4 (4.4) |  |
| Housewife/househusband | 876 (36.2) | 511 (31.1) | 483 (31.9) | 106 (28.6) | 40 (28.6) | 27 (30.0) |  |
| Part-time employed | 197 (8.1) | 122 (7.4) | 126 (8.3) | 28 (7.6) | 17 (12.1) | 6 (6.7) |  |
| Unemployed | 744 (30.7) | 556 (33.8) | 500 (33.0) | 134 (36.2) | 47 (33.6) | 42 (46.7) |  |
| Others | 223 (9.2) | 149 (9.1) | 187 (12.4) | 42 (11.4) | 11 (7.9) | 6 (6.7) |  |
| Degree of obesity, *n* (%) ^a^ |  |  |  |  |  |  |  |
| Underweight | 180 (7.4) | 102 (6.2) | 111 (7.3) | 20 (5.4) | 11 (7.9) | 10 (11.1) | 0.801 |
| Normal | 1791 (74.0) | 1204 (73.2) | 1119 (74.0) | 272 (73.7) | 100 (71.4) | 68 (75.6) |  |
| Obesity (class I) | 408 (16.9) | 309 (18.8) | 256 (16.9) | 74 (20.1) | 26 (18.6) | 9 (10.0) |  |
| Obesity (class II) | 37 (1.5) | 26 (1.6) | 24 (1.6) | 3 (0.8) | 3 (2.1) | 3 (3.3) |  |
| Obesity (class III) | 4 (0.2) | 3 (0.2) | 2 (0.1) | 0 (0.0) | 0 (0.0) | 0 (0.0) |  |
| Obesity (class IV) | 1 (0.0) | 0 (0.0) | 1 (0.1) | 0 (0.0) | 0 (0.0) | 0 (0.0) |  |
| Medical history of parents (with), *n* (%) |  |  |  |  |  |  |  |
| Stroke  IHDs | 532 (22.0)  278 (11.5) | 342 (20.8)  203 (12.3) | 311 (20.5)  172 (11.4) | 82 (22.2)  41 (11.1) | 40 (28.6)  20 (14.3) | 15 (16.7)  10 (11.1) | 0.214  0.848 |
| Smoking status, *n* (%) |  |  |  |  |  |  |  |
| Current/every day | 250 (10.3) | 174 (10.6) | 179 (11.8) | 24 (6.5) | 11 (7.9) | 14 (15.6) | 0.005 |
| Current/sometimes | 22 (0.9) | 13 (0.8) | 17 (1.1) | 6 (1.6) | 2 (1.4) | 1 (1.1) |  |
| Former | 712 (29.4) | 532 (32.3) | 484 (32.0) | 132 (35.7) | 54 (38.6) | 37 (41.1) |  |
| Never | 1438 (59.4) | 926 (56.3) | 834 (55.1) | 208 (56.2) | 73 (52.1) | 38 (42.2) |  |
| Drinking habits, *n* (%) |  |  |  |  |  |  |  |
| Once or more a week | 1084 (44.8) | 852 (51.8) | 730 (48.2) | 179 (48.4) | 69 (49.3) | 48 (53.3) | <0.001 |
| Less than once a week or never | 1338 (55.2) | 793 (48.2) | 784 (51.8) | 191 (51.6) | 71 (50.7) | 42 (46.7) |  |
| Regular exercise, *n* (%) |  |  |  |  |  |  |  |
| Current/always  Current/sometimes  Former  Never | 866 (35.8)  1004 (41.5)  307 (12.7)  245 (10.1) | 627 (38.1)  672 (40.9)  198 (12.0)  148 (9.0) | 534 (35.3)  656 (43.3)  177 (11.7)  147 (9.7) | 126 (34.1)  156 (42.2)  45 (12.2)  43 (11.6) | 42 (30.0)  68 (48.6)  17 (12.1)  13 (9.3) | 34 (37.8)  31 (34.4)  11 (12.2)  14 (15.6) | 0.485 |
| Sufficiency of sleep duration, *n* (%) |  |  |  |  |  |  |  |
| Short  Average  Good  Long | 632 (26.1)  238 (9.8)  1512 (62.4)  40 (1.7) | 415 (25.2)  141 (8.6)  1048 (63.7)  41 (2.5) | 376 (24.8)  159 (10.5)  937 (61.9)  42 (2.8) | 101 (27.3)  38 (10.3)  220 (59.5)  11 (3.0) | 44 (31.4)  18 (12.9)  71 (50.7)  7 (5.0) | 42 (46.7)  6 (6.7)  35 (38.9)  7 (7.8) | <0.001 |
| Depth of sleep, *n* (%) |  |  |  |  |  |  |  |
| Deep  Fairly deep  Average  Fairly light  Light | 496 (20.5)  1294 (53.4)  58 (2.4)  463 (19.1)  111 (4.6) | 309 (18.8)  931 (56.6)  26 (1.6)  307 (18.7)  72 (4.4) | 245 (16.2)  820 (54.2)  52 (3.4)  327 (21.6)  70 (4.6) | 52 (14.1)  187 (50.5)  11 (3.0)  96 (25.9)  24 (6.5) | 10 (7.1)  71 (50.7)  7 (5.0)  47 (33.6)  5 (3.6) | 8 (8.9)  36 (40.0)  6 (6.7)  26 (28.9)  14 (15.6) | <0.001 |
| Proximity of housing to the main road, *n* (%) |  |  |  |  |  |  |  |
| Along with road  ≤50 m  50 to ≤150 m  150 to ≤500 m  >500 m | 319 (13.2)  450 (18.6)  455 (18.8)  655 (27.0)  543 (22.4) | 213 (12.9)  285 (17.3)  351 (21.3)  436 (26.5)  360 (21.9) | 185 (12.2)  265 (17.5)  272 (18.0)  437 (28.9)  355 (23.4) | 41 (11.1)  53 (14.3)  77 (20.8)  107 (28.9)  92 (24.9) | 13 (9.3)  26 (18.6)  25 (17.9)  41 (29.3)  35 (25.0) | 5 (5.6)  18 (20.0)  18 (20.0)  28 (31.1)  21 (23.3) | 0.407 |
| Visibility of the main road, *n* (%) |  |  |  |  |  |  |  |
| Good  Partly  Poor | 285 (11.8)  602 (24.9)  1535 (63.4) | 191 (11.6)  403 (24.5)  1051 (63.9) | 177 (11.7)  370 (24.4)  967 (63.9) | 38 (10.3)  73 (19.7)  259 (70.0) | 16 (11.4)  37 (26.4)  87 (62.1) | 4 (4.4)  27 (30.0)  59 (65.6) | 0.297 |
| Bedroom is close to main road (with), *n* (%) | 306 (12.6) | 231 (14.0) | 213 (14.1) | 53 (14.3) | 24 (17.1) | 12 (13.3) | 0.528 |
| Duration of residence, *n* (%) |  |  |  |  |  |  |  |
| <3 years  3 to <20 years  ≥ 20 years | 133 (5.5)  780 (32.2)  1509 (62.3) | 71 (4.3)  537 (32.6)  1037 (63.0) | 54 (3.6)  449 (29.7)  1011 (66.8) | 9 (2.4)  111 (30.0)  250 (67.6) | 5 (3.6)  31 (22.1)  104 (74.3) | 5 (5.6)  23 (25.6)  62 (68.9) | 0.003 |
| Housing structure, *n* (%) |  |  |  |  |  |  |  |
| Wooden  Reinforced concrete  Others | 1438 (59.4)  842 (34.8)  142 (5.9) | 979 (59.5)  573 (34.8)  93 (5.7) | 926 (61.2)  513 (33.9)  75 (5.0) | 251 (67.8)  98 (26.5)  21 (5.7) | 91 (65.0)  38 (27.1)  11 (7.9) | 55 (61.1)  28 (31.1)  7 (7.8) | 0.072 |
| Use of an unvented-heating appliance in winter (with), *n* (%) | 1123 (46.4) | 772 (46.9) | 677 (44.7) | 170 (45.9) | 73 (52.1) | 38 (42.2) | 0.509 |
| Use of incense in the house (with), *n* (%) | 1273 (52.6) | 888 (54.0) | 828 (54.7) | 196 (53.0) | 78 (55.7) | 43 (47.8) | 0.642 |

^a^ Data were missing for four participants. ^b^ The six levels were (1) no noise, (2) not annoyed, (3) less annoyed, (4) moderately annoyed, (5) annoyed, and (6) highly annoyed. Abbreviations: IHD, ischemic heart diseases. ^c^ *P* value from χ^2^ test.
