# Supplementary material for: Association between environmental noise and subjective symptoms related to cardiovascular diseases among elderly individuals in Japan
Source: PLoS One. 2017 Nov 16;12(11):e0188236. doi: 10.1371/journal.pone.0188236 (PMC5690640; doi:10.1371/journal.pone.0188236)
Supplement: S2 Table — (DOCX) [file pone.0188236.s002.docx]

Online supporting information for the following article published in *PLoS ONE*

**Association between environmental noise and subjective symptoms related to cardiovascular diseases among elderly individuals in Japan**

Kenichi Azuma^1,2^, KIwao Uchiyama^2^

*1 Corresponding Author: Department of Environmental Medicine and Behavioral Science, Kindai University Faculty of Medicine, Osakasayama, Osaka, Japan*

*2 Sick-house Medical Science Laboratory, Division of Basic Research, Louis Pasteur Center for Medical Research, Kyoto, Japan*

**Table S2.** Correlation between sources of environmental noise annoyance.

|  | Automobile (d) | Automobile (n) | Neighborhood (d) | Neighborhood (n) | Construction (d) | Construction (n) | Railway (d) | Railway (n) | Aircraft (d) | Aircraft (n) |
| --- | --- | --- | --- | --- | --- | --- | --- | --- | --- | --- |
| Automobile (d) | 1.000 |  |  |  |  |  |  |  |  |  |
| Automobile (n) | **0.889^*^** | 1.000 |  |  |  |  |  |  |  |  |
| Neighborhood (d) | 0.571 | 0.560 | 1.000 |  |  |  |  |  |  |  |
| Neighborhood (n) | 0.548 | 0.555 | **0.908^*^** | 1.000 |  |  |  |  |  |  |
| Construction (d) | 0.510 | 0.524 | 0.581 | 0.585 | 1.000 |  |  |  |  |  |
| Construction (n) | 0.500 | 0.513 | 0.589 | 0.606 | **0.794^*^** | 1.000 |  |  |  |  |
| Railway (d) | 0.326 | 0.314 | 0.410 | 0.397 | 0.334 | 0.386 | 1.000 |  |  |  |
| Railway (n) | 0.318 | 0.312 | 0.403 | 0.393 | 0.329 | 0.379 | **0.970^*^** | 1.000 |  |  |
| Aircraft (d) | 0.342 | 0.345 | 0.375 | 0.372 | 0.370 | 0.367 | 0.418 | 0.417 | 1.000 |  |
| Aircraft (n) | 0.350 | 0.363 | 0.388 | 0.395 | 0.371 | 0.409 | 0.448 | 0.449 | **0.903^*^** | 1.000 |

Data are expressed as Spearman rank-correlation coefficients. *n* = 6,181. High correlations are denoted with asterisks at * r ≥ 0.7 in bold numbers for multicollinearity. All correlations are *p* < 0.01. Abbreviations: d, daytime; n, nighttime.
